# FRL and DAAM are required for lateral adhesion of interommatidial cells and patterning of the retinal floor

**DOI:** 10.1242/dev.201713

**Published:** 2023-11-24

**Authors:** Gabriella Gazsó-Gerhát, Rita Gombos, Krisztina Tóth, Péter Kaltenecker, Szilárd Szikora, Judit Bíró, Enikő Csapó, Zoltán Asztalos, József Mihály

**Affiliations:** ^1^ Institute of Genetics, HUN-REN Biological Research Centre, Temesvári krt. 62, Szeged H-6726, Hungary; ^2^ Doctoral School in Biology, Faculty of Science and Informatics, University of Szeged, Szeged H-6726, Hungary; ^3^ Aktogen Hungary Ltd., Szeged H-6726, Hungary; ^4^ Institute of Biochemistry, HUN-REN Biological Research Centre, Szeged H-6726, Hungary; ^5^ Department of Genetics, University of Szeged, Szeged H-6726, Hungary

**Keywords:** Compound eye, *Drosophila*, Formins, Interommatidial cells, DAAM, FRL

## Abstract

Optical insulation of the unit eyes (ommatidia) is an important prerequisite of precise sight with compound eyes. Separation of the ommatidia is ensured by pigment cells that organize into a hexagonal lattice in the *Drosophila* eye, forming thin walls between the facets. Cell adhesion, mediated by apically and latero-basally located junctional complexes, is crucial for stable attachment of these cells to each other and the basal lamina. Whereas former studies have focused on the formation and remodelling of the cellular connections at the apical region, here, we report a specific alteration of the lateral adhesion of the lattice cells, leaving the apical junctions largely unaffected. We found that DAAM and FRL, two formin-type cytoskeleton regulatory proteins, play redundant roles in lateral adhesion of the interommatidial cells and patterning of the retinal floor. We show that formin-dependent cortical actin assembly is crucial for latero-basal sealing of the ommatidial lattice. We expect that the investigation of these previously unreported eye phenotypes will pave the way toward a better understanding of the three-dimensional aspects of compound eye development.

## Teaser

**Summary:** Two formin proteins, FRL and DAAM, are required for the sealing of the interommatidial cell lattice necessary for optical insulation of the photoreception units in the insect compound eye.

## INTRODUCTION

The *Drosophila* compound eye comprises about 750-800 unit eyes (ommatidia) arranged into a highly stereotypic honeycomb pattern. Each ommatidium is composed of eight photoreceptor cells (RCs), four lens-secreting cone cells (CCs) and two primary pigment cells (PPCs). Neighboring ommatidia are separated from each other by the interommatidial cells (IOCs), including the secondary and tertiary pigment cells (SPCs and TPCs) as well as the bristle cell (BC) complexes located in every other vertex of the hexagonal lattice formed by the IOCs (Cagan and Ready, 1989). Whereas the central cell clusters function as independent photoreception units, the IOC lattice is required for optical insulation of the facets.

Eye development commences at the end of the larval stages when the monolayer of epithelial cells in the eye-antennal imaginal disc begins to differentiate (Wolff and Ready, 1991). RCs, forming a central cluster within the ommatidia, are the first cells to acquire their cell fate and become sensory neurons projecting axons into the optic lobes. Following the RCs, four non-neuronal CCs are added to the cluster and, subsequently, two PPCs are recruited that encircle the whole cluster. Recruitment of these central cells is completed by about 20 h after puparium formation (APF) (Cagan and Ready, 1989). During the next 20-24 h, undifferentiated cells between the ommatidial clusters also undergo differentiation, resulting in the formation of an interommatidial lattice, where SPCs form the sides of the hexagons, whereas TPCs and BCs are located at the vertices. By 40-44 h APF, the basic cellular pattern of the retina is completed and it reaches a thickness of 30 µm, exhibiting a characteristic apical-basal pattern (Cagan and Ready, 1989; Longley and Ready, 1995). At the level of the apical adherens junctions (AJs), presence of the four CCs, enwrapped by the two PPCs, is evident. These clusters are separated from each other by six SPCs, three TPCs and three BCs, arranged in a highly stereotypic manner. Importantly, the RCs acquire a more basal position and they are not detectable on the apical sections. Conversely, lateral sections of the eye are devoid of CCs and PPCs, but the position of the RCs becomes evident, together with the IOCs surrounding the RCs. Compared to these layers, the level of the retinal floor displays a peculiar rosette or flower petal pattern composed of the flattened endfeet of the IOCs and the axonal exit sites (Longley and Ready, 1995). To achieve perfect optical shielding, the basal feet of the SPCs and TPCs move under the centrally located RCs and CCs, and gradually flatten into thin plates, which form the retinal floor together with the underlying basal lamina (Longley and Ready, 1995). Axon bundles of the RCs pass through the retinal floor below each ommatidium at holes surrounded by the grommets of the basement-membrane extracellular matrix, serving as focal adhesion (FA) sites for the IOC feet.

Previous research has successfully identified the molecules of cell fate specification (Kumar, 2012) and much has been learnt about the mechanisms of tissue patterning through AJ and cytoskeleton remodeling (Del Signore et al., 2018; Founounou et al., 2021; Johnson, 2020; Johnson, 2021). For example, DE-cadherin (Shg) and N-cadherin (CadN) were shown to be required for apical adhesion between CCs, as well as for ommatidial rotation (Hayashi and Carthew, 2004; Mirkovic and Mlodzik, 2006). Members of the immunoglobulin superfamily adhesion molecules, such as Hibris (Hbs), Roughest (Rst), Sticks and stones (Sns) and Kin of Irre (Kirre), are necessary for preferential adhesion and sorting of the IOCs (Araujo et al., 2003; Bao and Cagan, 2005; Bao et al., 2010; Grzeschik and Knust, 2005; Johnson et al., 2012; Larson et al., 2008; Reiter et al., 1996); the adaptor protein Cindr was implicated in linking junction and actin cytoskeleton regulation (Johnson et al., 2012, 2011, 2008), and cofilin (ADF or Tsr) is crucial for retinal elongation and rhabdomere morphogenesis (Pham et al., 2008). Besides these factors, Rho1 is required to maintain the AJs of pigment cells by inhibiting the endocytosis of DE-cadherin in a Cdc42-dependent manner, and to maintain apical tension in a Rok/myosin II-dependent manner (Warner and Longmore, 2009a,b; Yashiro et al., 2014), whereas Rac signaling and myosin II are important for cell shape changes during lattice formation and IOC remodeling (Bruinsma et al., 2007; Del Signore et al., 2018). Because of the prime importance of AJs, these studies mostly focused on apical cell adhesion complexes and apical organization of the developing pupal eye. In addition, it has been revealed that integrins are required for retinal floor organization (Longley and Ready, 1995) and a recent study highlighted the importance of non-AJ membrane domains in cell shape changes in the retina, dictated by Hippo signaling and spectrins (Deng et al., 2020). Despite these studies, the factors and mechanisms contributing to morphogenesis of the lateral and basal regions of the retina remained largely unknown.

Here, we report that two formin-family cytoskeleton regulatory proteins, DAAM and FRL, play a redundant role in the patterning of the pupal eye. Whereas the *DAAM* and *frl* single mutants exhibited perfectly organized eyes resembling wild-type eyes, the concomitant loss of these formins resulted in a severe impairment of IOC lattice formation. In the absence of DAAM and FRL, the IOCs acquired an abnormal shape and position in the lateral and basal layers of the eye, whereas their apical AJ connections and organization were only mildly affected. Remarkably, lateral adhesion of the IOCs was often lost, and the IOCs were no longer able to separate the RCs of the neighboring ommatidia. Moreover, the axonal exit sites in the basal retina became irregularly spaced due to the aberrant shape and misplacement of the IOCs. In summary, we show that formin-dependent regulation of the IOC actin cytoskeleton is crucial for retinal floor organization and perfect sealing of the ommatidial lattice.

## RESULTS

### *DAAM*; *frl* double mutants exhibit a rough eye and fused ommatidia

While studying the potentially redundant and non-redundant developmental roles of two *Drosophila* Diaphanous-related formins (DRFs), namely, DAAM and FRL, we formerly found unique and overlapping roles in eye development and axonal growth (Dollar et al., 2016). To extend these studies, we decided to focus on two homozygous viable alleles: *DAAM^Ex4^*, a DAAM-PB isoform-specific allele (Gombos et al., 2015), and *frl^59^*, a recently created null allele (Dehapiot et al., 2020). We noticed that although eyes of the *DAAM^Ex4^* or *frl^59^* single mutants were wild type and the stocks were fully viable as adults, the double homozygous mutant combination was pharate adult lethal, with a few escapers exhibiting rough eyes.

**Fig. 1.**
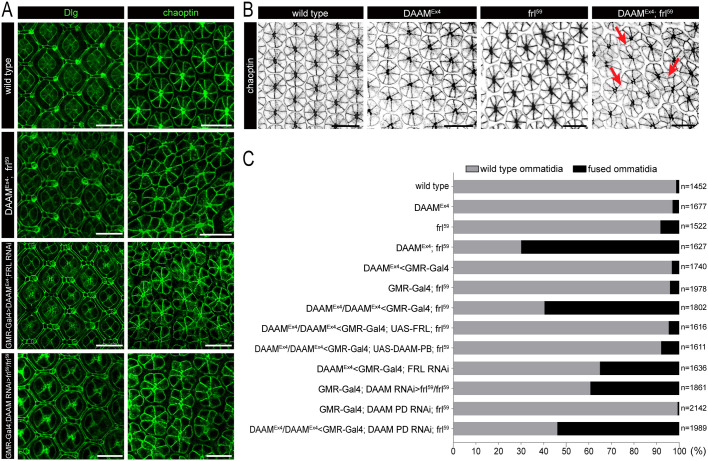
**Pupal eyes of the *DAAM*; *frl* double mutants exhibit ommatidia fusions.** (A) Confocal immunofluorescence images of pupal eyes at 48 h APF stained with anti-Dlg (left panels) and 24B10 (anti-chaoptin) (right panels) antibodies. Genotypes are indicated on the left. Note that in wild-type eyes, 24B10 labels the eight photoreceptor cells (RCs) of the ommatidial clusters, which are perfectly separated from each other. In contrast, the RC clusters often abut each other in the formin mutants. (B) 24B10 staining of *DAAM^Ex4^* and *frl^59^* single-mutant eyes at 48 h APF revealed no alterations compared with wild-type eyes, whereas in the double-mutant eye, several clusters appeared to touch each other (three examples are indicated by red arrows), which was designated as ommatidia fusion. (C) Quantification of the ommatidia fusions in pupal eyes of the indicated genotypes at 48 h APF. ‘*n*’ indicates the number of ommatidia counted. Data are representative of 13-16 animals per genotype. Scale bars: 10 µm.

To gain cellular level insights into the underlying defects, we analyzed the eyes after the early stages of pupal development at 48 h APF, by which point all retinal cells should already have been specified and patterned into a precise honeycomb lattice. First, we examined the RC clusters with the commonly used marker chaoptin (Chp). We found that, unlike in wild type, in the formin double mutants, many of the RC clusters failed to separate and RCs from the neighboring clusters often abutted each other (Fig. 1A,B). This is a previously unreported eye phenotype that we designated ‘ommatidia fusion’ for simplicity. The number of RC clusters involved in the fusion events varied from two to 15-20 depending on the region and the eye, most typically eight to ten clusters getting in contact with each other with some clusters abutting more than two neighbors (Fig. 1A,B). We quantified the ommatidia fusion phenotype by counting the number of RC clusters involved in any kind of a fusion event regardless of the size of the fused clusters, revealing that the penetrance of this phenotype was about 70% in *DAAM^Ex4^*; *frl^59^* double mutants (Fig. 1C). In further support of the redundant formin roles, eye-specific knockdown of *frl* in a *DAAM^Ex4^* mutant background (*DAAM^Ex4^*>*GMR-Gal4*; *frl^RNAi^*) also resulted in ommatidium fusion with a 35% penetrance (Fig. 1C). Similarly, concurrent knockdown of both major DAAM isoforms (DAAM-PB and DAAM-PD) in an *frl* mutant background (*GMR-Gal4*; *DAAM^RNAi^*; *frl^59^*) gave rise to the formation of fused ommatidia (about 39%) (Fig. 1C). In contrast to this, PD isoform-specific silencing of *DAAM* in an *frl* mutant did not impair eye development (Fig. 1C). The ommatidium fusion defects of *DAAM^Ex4^*; *frl^59^* could be fully rescued by eye-specific expression of *UAS-DAAM-PB* or *UAS-FRL* (containing the full-length *frl* cDNA) (Fig. 1C), which is in line with the phenotype of the single mutants. These results indicate that, of the two DAAM isoforms, only DAAM-PB is specifically required, and that DAAM-PB and FRL have largely, if not entirely, overlapping contributions to patterning during the first 48 h of pupal eye development.

### Apical organization of the pupal eye is mildly affected by the lack of DAAM and FRL

**Fig. 2.**
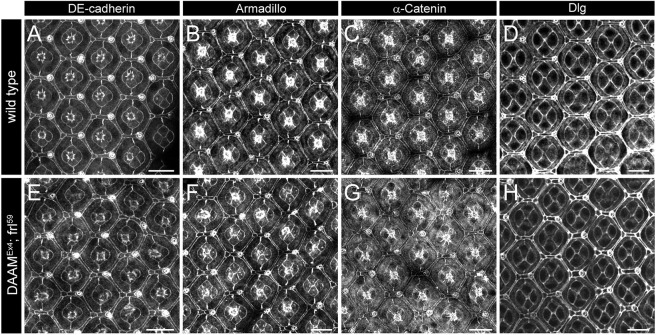
**DAAM and FRL are not required for apical accumulation of the major adherens and septate junction proteins.** (A-H) The apical accumulation of the AJ proteins DE-cadherin (A,E), Armadillo (B,F) and α-Catenin (C,G) seen in wild-type eyes (A-C) is not altered in *DAAM^Ex4^*; *frl^59^* double-mutant eyes (E-G) at 48 h APF, nor is distribution of the Dlg protein (D,H) marking the septate junctions. Images are representative of ten animals per genotype. Scale bars: 10 µm.

To further explore the defects observed in the formin double mutants, we initiated a systematic analysis of all other cell types of the eye. To begin with, we evaluated the apical region of the pupal retina, which was first assessed by DE-cadherin staining revealing the AJs of the CCs, PPCs and IOCs (Fig. 2A). Compared with wild-type eyes, the eyes of *DAAM^Ex4^*; *frl^59^* double mutants displayed several kinds of minor alterations, including shortening of the horizontal SPCs, eventual flips in the positions of the BCs and TPCs, and irregularities in the hexagonal arrangement (Fig. 2A,B). Despite these, overall organization of the apical region remained largely normal without an obvious change in DE-cadherin levels (Fig. 2A,E). Similarly, two additional AJ markers, Armadillo (Arm) and α-Catenin (α-Cat), also showed no significant differences in wild-type versus double-mutant eyes (Fig. 2B,C,F,G), nor did N-cadherin (Fig. S1), another major junctional protein expressed in the CCs and RCs. Moreover, we did not detect a noticeable alteration in localization of the septate junction protein Dlg (or Dlg1) (Fig. 2D,H). Next, we asked whether cell number was affected by the lack of DAAM and FRL, but the numbers of CCs, PPCs, SPCs (all marked by DE-cadherin) and RCs (marked by chaoptin) did not change in the mutants, whereas the number of TPCs was slightly higher at the expense of the number of BCs (Fig. S2). Thus, these data suggest that at the level of the two important junctional areas, the adherens and septate junctions, the major junctional proteins were present in the double mutants in similar amounts as in wild type, and it follows that overall patterning of the apical regions was mostly unaffected by the concomitant absence of DAAM and FRL.

### The loss of DAAM and FRL impairs basal patterning of the eye

**Fig. 3.**
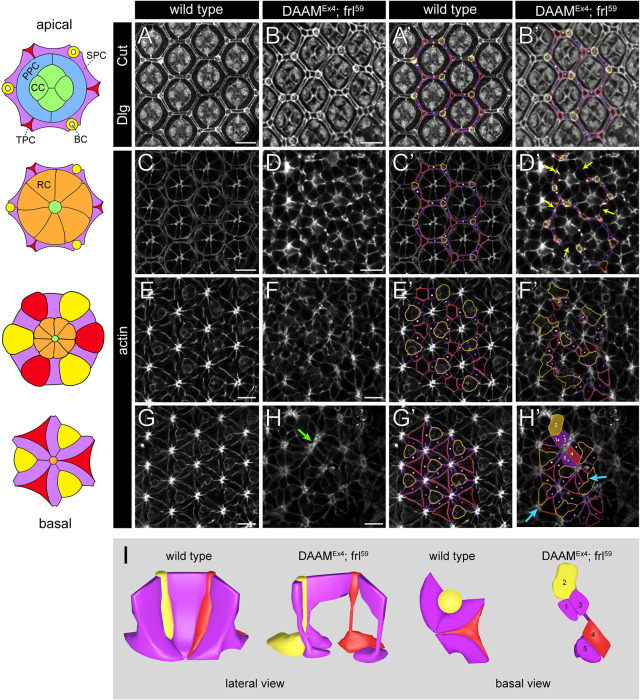
**DAAM and FRL are required for lateral attachment of the IOCs and patterning of the retinal floor.** (A-H′) Confocal *z*-sections of eyes with *54C-Gal4/UAS-mCD8-GFP* in a wild-type (control) or a *DAAM^Ex4^*; *frl^59^* mutant background at 48 h APF stained for Dlg and Cut (A-B′) and actin (C-H′), which were used to trace individual IOCs across the apical-basal axis of the eye (note that GFP is not shown here). Schematic drawings on the left indicate the wild-type ommatidial cell pattern at four positions along the apical-basal axis. Note that each cell is color coded, and that the IOCs [the six secondary pigment cells (SPCs), three tertiary pigment cells (TPCs) and three bristle cells (BCs)] at the apical (top row) and lateral (second row) layers of the eye appear as thin walls in between the clusters. At the basal layers (bottom rows), flattened feet of the IOCs become apparent, forming a flower petal pattern where the six SPCs link the axonal exit sites (located in the central position in each cluster) and separate the TPCs and BCs, located at alternating positions in between the petals. Panels A′-H′ show the same images as A-H, and in A′-H′, a group of IOCs, surrounding four ommatidia, are contoured using the same color code shown in the schematics on the left. In addition, the contoured SPCs are coded individually as well with colored dots and crosses. Note that at the septate junctions (marked by Dlg), the IOCs form a hexagonal lattice that is evident in the wild-type (A,A′) and formin mutant (B,B′) eye as well, although many of the horizontal SPCs appear shorter than normal in the mutant eye. Compared with this, at the lateral layer (C-D′), the IOCs often lose connection with each other (yellow arrows) and they often exhibit altered cell shapes, resulting in the formation of a broken lattice in the mutant eye. In the basal layers (E-H′), the shape of the IOCs is highly irregular in the formin mutants (F,F′,H,H′), and the TPCs and BCs occasionally make aberrant contacts with each other (blue arrows in H′), which is not observed in the wild-type eyes. The green arrow in H points to axonal exit sites erroneously contacting each other. Images are representative of ten animals per genotype. Scale bars: 10 µm. CC, cone cell; PPC, primary pigment cell; RC, photoreceptor cell. (I) Graphical 3D reconstructions of a group of IOCs consisting of three SPCs (in purple), one TPC (in red) and one BC (in yellow) illustrate the alterations in cell shape and cell contacts in wild-type versus formin mutant eyes. Note the discontinuities in the lattice wall (formed by the SPCs) due to impaired lateral adhesion between the SPCs and the corner cells, and the cell shape changes in the endfeet region. Reconstruction in the case of the mutant is based on the confocal *z*-sections of the cells numbered from 1 to 5 in H′.

Although analysis of the apical markers revealed that the position and number of the IOCs did not change significantly in the *DAAM^Ex4^*; *frl^59^* mutant eyes, it appeared that the IOCs were no longer able to insulate the RCs in the lateral region of the eye, implying that the lattice organization was at least partly lost. To test this directly, we expressed mCD8::GFP in the developing IOCs by *54C-Gal4* (a driver specific for the SPCs and TPCs) (Nagaraj and Banerjee, 2007) (Fig. S3) and labeled the BCs and CCs by Cut (Ct) staining while visualizing the cell borders with phalloidin. This approach allowed us to follow the shape of the IOCs from the top to the bottom of the retina. After confocal *z*-sectioning across the entire apical-basal axis of a wild-type retina, we could clearly detect the typical IOC pattern in the apical, lateral and basal layers (Fig. 3). By contrast, although apical IOC organization looked largely normal (Fig. 3A-B′), the shape and position of the IOCs in the lateral and basal regions were severely impaired in the formin double mutants (Fig. 3C-H′). The most prominent change in the lateral region was the loss of the continuous lattice, as many gaps were detected in the walls of the hexagons due to misplacement and shape changes of the IOCs (Fig. 3D′,I) (Fig. S3). By tracing individual IOCs across the retina, it was obvious that the IOCs failed to maintain their lateral contacts with each other, leaving space for RCs of the neighboring ommatidia to make aberrant contacts (Fig. 3D′,F′,I). Thus, these observations strongly suggest that below the apical layers of the eye, DAAM and FRL are required for maintaining the shape and lateral adhesion of the IOCs.

**Fig. 4.**
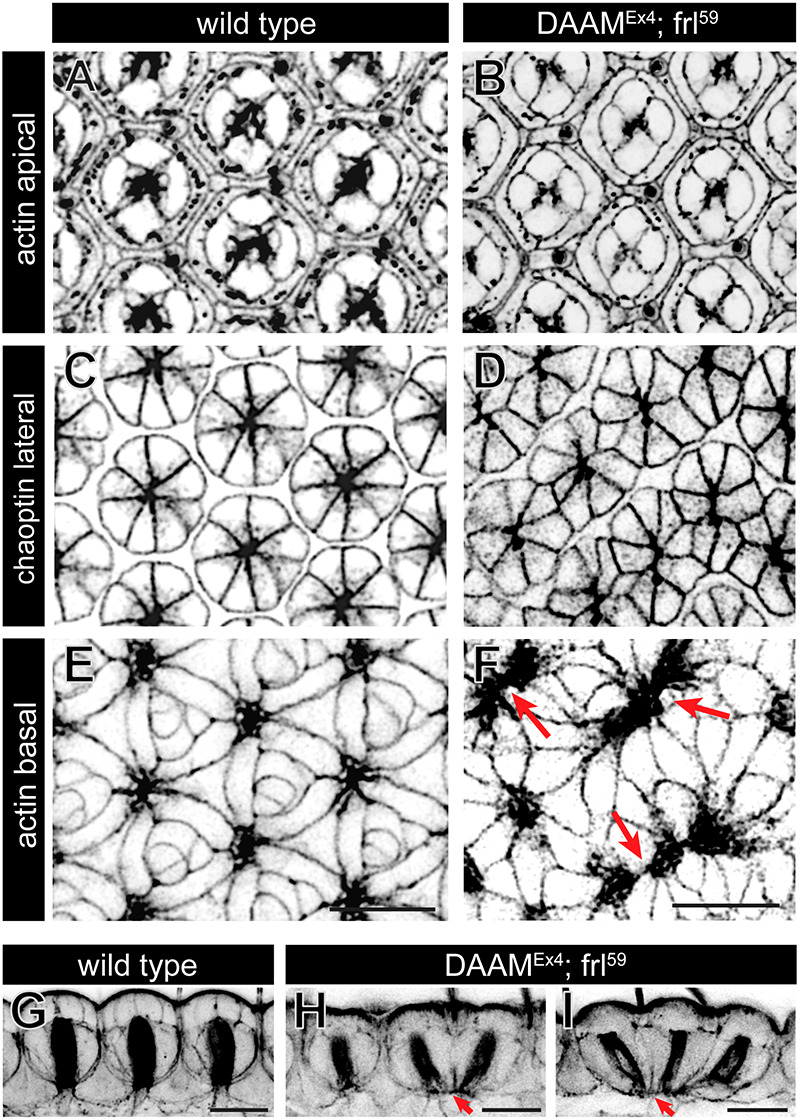
**The lateral and basal patterning defects in *DAAM*; *frl* double-mutant eyes.** (A-F) Confocal *z*-sections of a wild-type (A,C,E) and a *DAAM^Ex4^*; *frl^59^* double-mutant eye (B,D,F) at 48 h APF stained for actin (A,B,E,F) and chaoptin (C,D). Although the apical pattern was largely normal in the formin mutant (compare A with B), the lateral section revealed the fused ommatidial clusters (C,D), and in the basal section, the irregularly spaced axonal exit sites are evident (arrows in F). (G-I) Sagittal pupal eye sections of a wild-type (G) and two *DAAM^Ex4^*; *frl^59^* double-mutant (H,I) eyes stained for actin. Dense staining of the rhabdomeres marks the main axis of the facets and indicates the basal axonal exit sites, which are regularly spaced in the wild-type eye (G). Note the distorted rhabdomere orientation and irregular spacing of the exit sites (red arrows) in the formin double mutants (H,I). Images are representative of ten animals per genotype. Scale bars: 10 μm.

The basal part of the honeycomb lattice in the eye is formed by the IOC feet, exhibiting a flower petal pattern and the regularly arranged axonal exit sites (Figs 3G,G′ and 4A-F). In the *DAAM^Ex4^*; *frl^59^* double mutants, this highly ordered basal pattern (Longley and Ready, 1995), typical for wild-type eyes, was lost to a large extent. Most notably, the position of the axonal exit sites became uneven with some sites nearly touching each other, which resulted in the distortion of the hexagonal array at many places (Figs 3H,H′ and 4F). The SPCs were often misplaced in the array and many of them failed to establish stable contacts with two exit sites and to function as a proper spacer between them (Fig. 3H′). In addition, the SPCs also failed to separate TPCs and the BC complexes, which never contact in wild-type situations (Fig. 3G′,H′). Overall, the shape of most IOC feet appeared abnormal and the rosette pattern of the retinal floor vanished (Fig. 4F). These robust changes could also be appreciated on sagittal sections where the future rhabdomeres mark the main axis of the facets (Fig. 4G-I). Contrasting to the highly regular wild-type rhabdomere distribution, the mutant eyes were characterized by abnormally spaced rhabdomeres (often with a distorted orientation compared with that in wild-type ommatidia), altered cell shape and axonal exit positions (Fig. 4G-I).

Integrins were shown to play an important role in attaching the IOC feet to the basal lamina and to the grommets in the retinal floor (Longley and Ready, 1995). In wild-type eyes, an anti-β_PS_-integrin (Mys) antibody highlights the FAs at the grommets and along the contacts between the pigment cell feet (Fig. S4A). Upon the loss of DAAM and FRL, a similarly strong Mys staining compared to that in controls was evident at most of the grommets and along the IOC feet (Fig. S4B). As an additional control, we also examined the distribution of Talin (Rhea), another important FA protein (Klapholz and Brown, 2017). As expected, Talin was accumulated at the grommets and in the IOC feet without exhibiting a clear difference in its expression levels and pattern in the wild-type versus mutant eyes (Fig. S4C,D). However, irregular spacing of the axonal exit sites was visible with both FA markers and some grommets had an unusually extended shape, whereas some others exhibited a lower level of staining than in wild-type. Based on these observations, most FA sites were present in the formin double mutants, yet a significant part of the grommets looked abnormal either because they failed to form a perfect circle around the exit sites or because they fused with each other (Fig. S4). Besides the defects in grommet formation and arrangement, the irregular shape and position of the lattice cell feet were also obvious (Fig. S4).

Collectively, these data revealed a crucial role for DAAM and FRL in patterning the retinal floor by determining the shape of the IOC feet and the grommet attachment of the SPCs. Although basal membrane attachment of the IOC feet did not appear to be affected in the absence of these formins, lateral adhesion of the IOCs clearly depends on formin function.

### DAAM and FRL are required in the IOCs

**Fig. 5.**
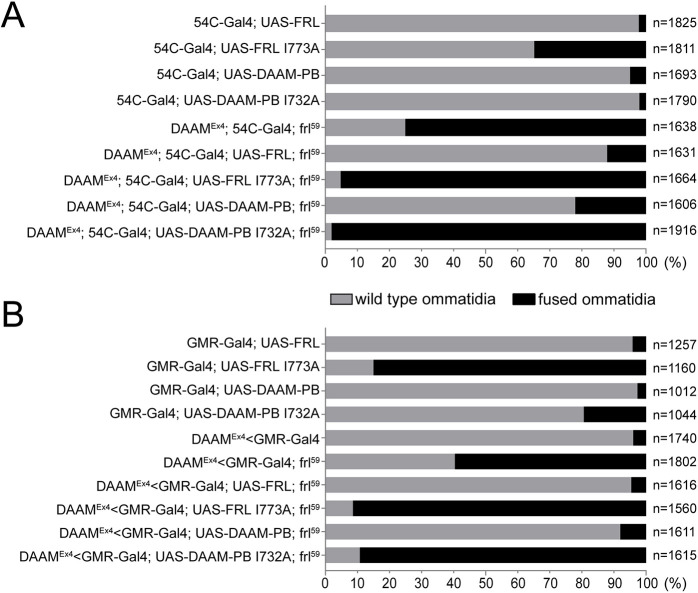
**Rescue of the ommatidia fusion with wild-type and actin polymerization-incompetent formins.** (A,B) Quantification of the ommatidia fusion phenotypes in pupal eyes of the genotypes indicated. IOC-specific (*54C-Gal4*-driven) (A) and eye-specific (*GMR-Gal4*-driven) (B) expression of the wild-type FRL or DAAM-PB isoform was able to rescue the ommatidium fusion phenotype of the formin double mutants to a large extent, although *GMR-Gal4* provided a somewhat better rescue than *54C-Gal4*. Conversely, the mutant forms impaired in actin polymerization (FRL-I773A and DAAM-PB I732A) did not rescue at all; instead, they enhanced the fusion phenotype, presumably by a dominant-negative effect also exhibited upon their expression in a wild-type background. ‘*n*’ indicates the number of ommatidia counted. Data are representative of 10-14 animals per genotype.

The ommatidium fusion phenotype and the retinal floor defects observed in the *DAAM^Ex4^*; *frl^59^* mutants indicated a formin requirement in the IOCs. To test whether DAAM and FRL functions are only needed in the IOCs, we used the pigment cell-specific *54C-Gal4* driver. When *UAS-DAAM-PB* or *UAS-FRL* expression was controlled by *54C-Gal4* in a formin double-mutant background, the ommatidia fusion phenotype was rescued in 78% and 88% of the unit eyes, respectively (Fig. 5A). Whereas *GMR-Gal4*, expressed in all cells of the retina, provided a somewhat better rescue with 92% for DAAM-PB and 95% for FRL (Fig. 5B), we note that *54C-Gal4* is not active in the BCs and, therefore, these data support a primary formin requirement in the IOCs. Consistent with this possibility, knockdown of *frl* in a *DAAM^Ex4^* mutant background with *54C-Gal4* (*DAAM^Ex4^*; *54C-Gal4*; *frl^RNAi^*) resulted in similar eye phenotypes as typical for the double formin mutants (Fig. S5).

**Fig. 6.**
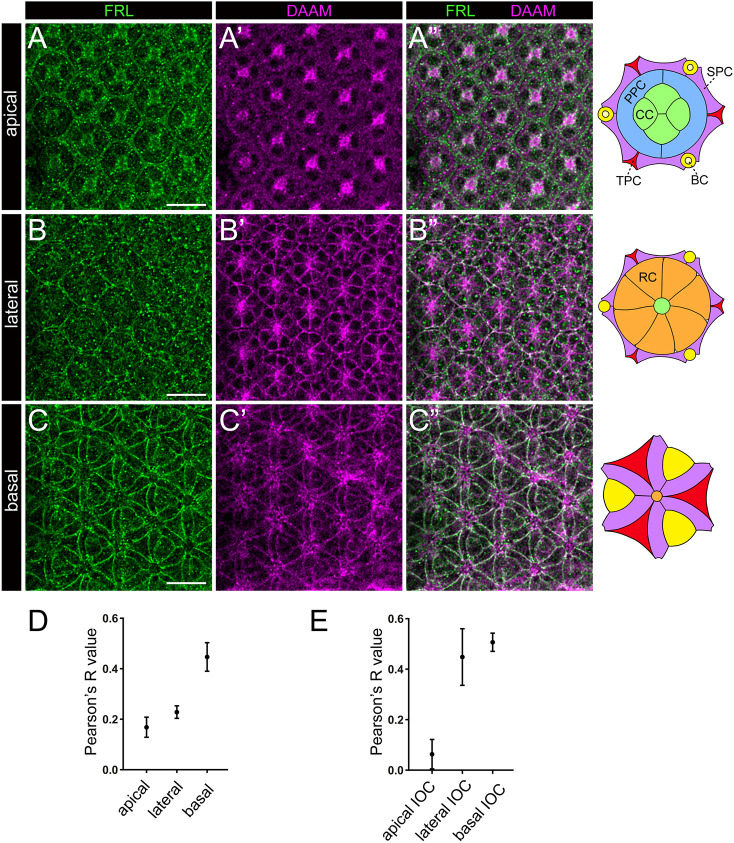
**Localization of DAAM and FRL in the pupal eye at 48 h APF.** (A-C″) Confocal *z*-sections reveal that in the apical region of the pupal eye (A-A″), FRL is enriched in the IOCs (mainly in the cell cortex) and in the cone cells (CCs) (A), whereas DAAM is present in the primary pigment cells (PPCs) and in the CCs (A′) without a significant overlap with FRL (A″). In the lateral region of the eye (B-B″), FRL is mainly visible in the cortical membrane of the IOCs (B), whereas DAAM shows an overlapping accumulation with FRL in the IOC cortex, and an enrichment in the photoreceptor cell (RC) membranes and in the axons (B-B″). At the basal region (C-C″), we detected a strong FRL and DAAM colocalization at the retinal floor along the cortical membrane of the IOCs (C-C″). In addition, several DAAM-positive foci were evident in the region of the axonal exit sites (C′). Images are representative of ten animals per genotype. Scale bars: 10 µm. Schematic drawings on the right indicate the wild-type ommatidial cell pattern at three positions along the apical-basal axis. BC, bristle cell complex; SPC, secondary pigment cell; TPC, tertiary pigment cell. (D,E) Quantification of the degree of FRL and DAAM colocalization in the apical, lateral and basal layers of the eye for all retinal cells (D) and for the IOCs (E). Data show the mean±s.d.

To further corroborate these findings, we examined the protein expression pattern of DAAM and FRL in the developing pupal eye. The specificity of the anti-DAAM antibody has already been reported (Gombos et al., 2015), whereas the recently generated anti-Frl antibody (Toth et al., 2022) exhibits a specific membrane enrichment in the retinal cells, together with some non-specific background in the cytoplasm of the CCs and RCs (Fig. S6). At 48 h APF, we detected a strong FRL accumulation in the retinal floor along the cortical membrane of the IOCs (Fig. 6C,C″), and a similar pattern was evident in the case of DAAM as well (Fig. 6C′). Interestingly, the FRL staining was uniformly strong along the borders of the individual IOC feet, but FRL did not accumulate at the circles of the grommets (Fig. 6C). DAAM showed no clear grommet association either, although several DAAM-positive foci were evident in the region of the axonal exit sites (corresponding to the RC axons as judged by the analysis of multiple optical sections) (Fig. 6C′). In the lateral region of the eye, FRL was mainly found in the cortical membrane of the IOCs (as indicated by their cell shape), whereas DAAM showed a partly overlapping accumulation, together with an enrichment in the RC membranes and in the axons (Fig. 6B-B″). On apical optical sections, we detected a largely uniform FRL accumulation in the cytoplasm (and presumably in the cortical membrane) of the IOCs and a strong signal in the CCs, whereas DAAM was present in the PPCs and in the CCs (Fig. 6A-A″), without a significant overlap with FRL in the IOCs. In line with these observations, quantification of the level of colocalization in all retinal cells (Fig. 6D) or in the IOCs (Fig. 6E) revealed strong colocalization in the basal layers, moderate colocalization in lateral layers and weak colocalization in the apical sections.

Based on these findings, we conclude that DAAM and FRL affect eye patterning by playing a cell-autonomous role in the IOCs. Consistent with this and the distinct effects along the apical-basal axis, both formins are enriched in the basal and lateral regions of the IOC membranes, but they fail to show a notable overlay in their apical distribution. This differential apical expression pattern taken together with the lack of eye phenotypes in the *DAAM* or *frl* single mutants raise the possibility that the apical role of these formins is redundant with other members of the family.

### The actin assembly activity of DAAM and FRL is essential for lattice formation

Although many formins were linked to microtubule regulation and they have been implicated in actin/microtubule crosslinking, the formin type of proteins are best known for their ability to promote actin assembly (Chesarone et al., 2010). To address whether DAAM and FRL are involved in the regulation of the actin cytoskeleton in the pupal retina, we tested their actin polymerization-incompetent mutant forms (*UAS-FLDAAM-I732A* and *UAS-FRL-I773A*) (Molnar et al., 2014). These point mutations are equivalent to the I845A mutation of the mouse formin mDia1 (encoded by *Diap1*) and the I1431A mutation of the yeast formin Bni1, which were previously shown to disrupt actin binding and assembly *in vitro* (Harris et al., 2006; Lu et al., 2007; Xu et al., 2004). In contrast to the wild-type formin transgenes, neither *FLDAAM-I732A* nor *FRL-I773A* was able to rescue the ommatidium fusion phenotype of *DAAM^Ex4^*; *frl^59^* when expressed with *GMR-Gal4* or *54C-Gal4* (Fig. 5A,B). Thus, these results indicate that the actin nucleation and polymerization activity of DAAM and FRL are necessary for IOC development and, similar to our previous findings for *FLDAAM-I732A* in the adult brain (Gombos et al., 2015), the mutant transgenes exhibited a moderately strong dominant-negative effect (Fig. 5A,B).

**Fig. 7.**
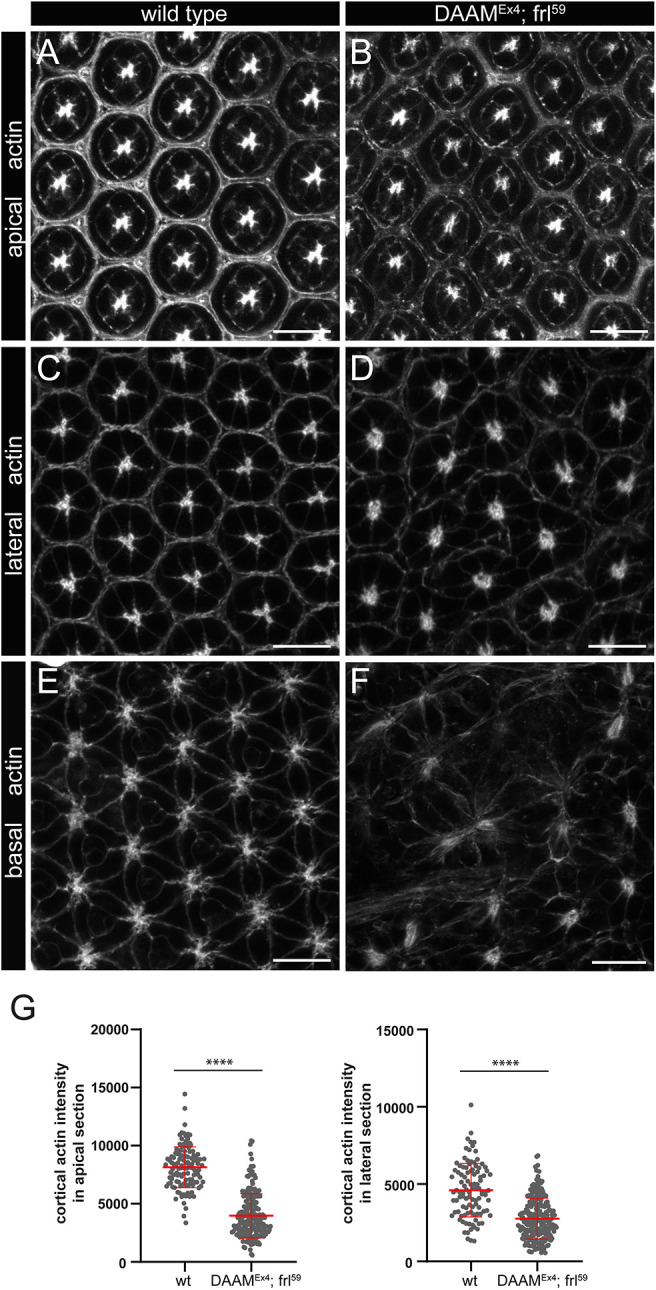
***DAAM^Ex4^; frl^59^* double-mutant eyes exhibit reduced cortical actin levels in IOCs.** (A-D) Phalloidin staining of *DAAM^Ex4^; frl^59^* double-mutant eyes revealed reduced actin levels along the cortical cell membranes of the IOCs in the apical (A,B) and lateral sections (C,D) of the eye compared with those in wild-type controls (quantified in G). (E,F) Although a similar actin intensity analysis was not feasible in the endfeet region because the cell shape in the double mutants is highly irregular (F), the basal cortical actin signal clearly appeared more diffuse and weaker in the formin mutants than in the wild type (E). Scale bars: 10 µm. (G) Quantification of actin intensity along the cortical membrane of the IOCs on apical and lateral confocal *z*-sections in wild-type (wt) and formin mutant eyes. Data show the mean±s.d. and are representative of ten animals per genotype. ****P<0.0001 (two-tailed, unpaired Mann–Whitney test).

Former studies revealed the importance of F-actin in AJ formation and, accordingly, several actin-regulatory proteins, such as Cdc42, Rho1, Cindr and spectrins, were linked to junctional actin regulation in the pupal fly eye (Deng et al., 2020; Johnson et al., 2008; Warner and Longmore, 2009a,b). Moreover, it was found that during 22-42 h APF, the membrane and AJ-associated F-actin undergo dynamic rearrangements, and that numerous cytoplasmic actin bundles, emanating from the AJs, also form in the apical region of the IOCs (Del Signore et al., 2018; Johnson et al., 2008). To explore the role of DAAM and FRL in actin organization, we analyzed apical actin organization in fixed and live samples with phalloidin staining and LifeAct::GFP, respectively, in wild-type and *DAAM^Ex4^*; *frl^59^* double-mutant eyes. Whereas we found no significant differences in apical actin organization in the formin mutant eyes (Fig. S7), quantitative analysis revealed a decrease in the level of apical actin (Fig. 7A,B,G). Subsequently, the lateral and basal layers were investigated, where F-actin was mostly evident at the cortical cell membranes in a wild-type pupal retina at 48 h APF (Fig. 7C,E). Staining of the *DAAM^Ex4^*; *frl^59^* double-mutant eyes exhibited reduced average actin levels along the cortical cell membranes in the lateral sections (Fig. 7D,G), where weakly stained borders were often obvious. A similar analysis of the most basal, endfeet region was not feasible, because the cell shape and, consequently, the basal cortical actin pattern was highly irregular in the double mutants (Fig. 7F), preventing reliable measurements along the cortical membranes. Nevertheless, the basal cortical actin signal clearly looked less uniform, more diffused and overall weaker than in the wild type (Fig. 7E,F). Taken together, these findings indicate that, consistent with their expression pattern, DAAM and FRL appear to be required for cortical actin formation in all layers of the eye, yet they have a crucial role only in the lateral and basal region of the IOCs.

### Cdc42 and Zip work together with DAAM and FRL in latero-basal eye development

**Fig. 8.**
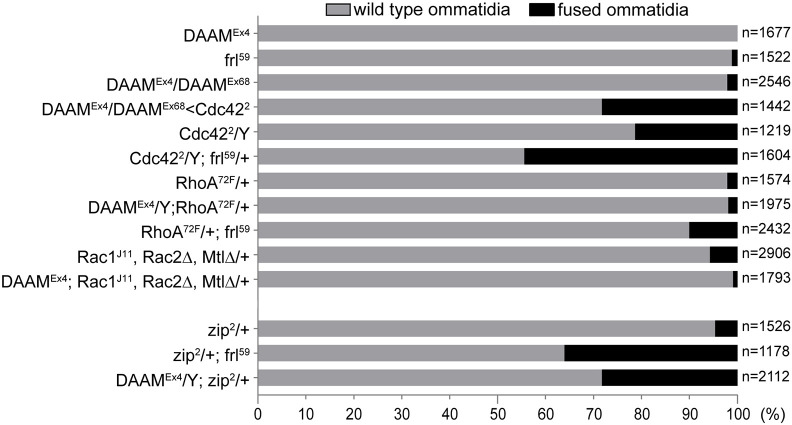
***DAAM* and *frl* show a dominant genetic interaction with *Cdc42* and *zip*.** Quantification of the ommatidia fusion defects revealed that heterozygosity for *Cdc42^2^* dominantly enhanced the mild phenotype of *DAAM^Ex4^*/*DAAM^Ex68^*. Likewise, the weak fusion phenotype of *Cdc42^2^* was enhanced by *frl^59^* heterozygosity. In contrast, *Rho1* and the triple Rac mutant (*Rac1^J11^*, *Rac2Δ*, *MtlΔ*) did not show a genetic interaction with *DAAM^Ex4^* or *frl^59^* single mutants. Moreover, *zip^2^* was also a dominant enhancer of the very mild fusion phenotype displayed by *DAAM^Ex4^* and *frl^59^*. ‘*n*’ indicates the number of ommatidia counted. Data are representative of 12-18 animals per genotype.

**Fig. 9.**
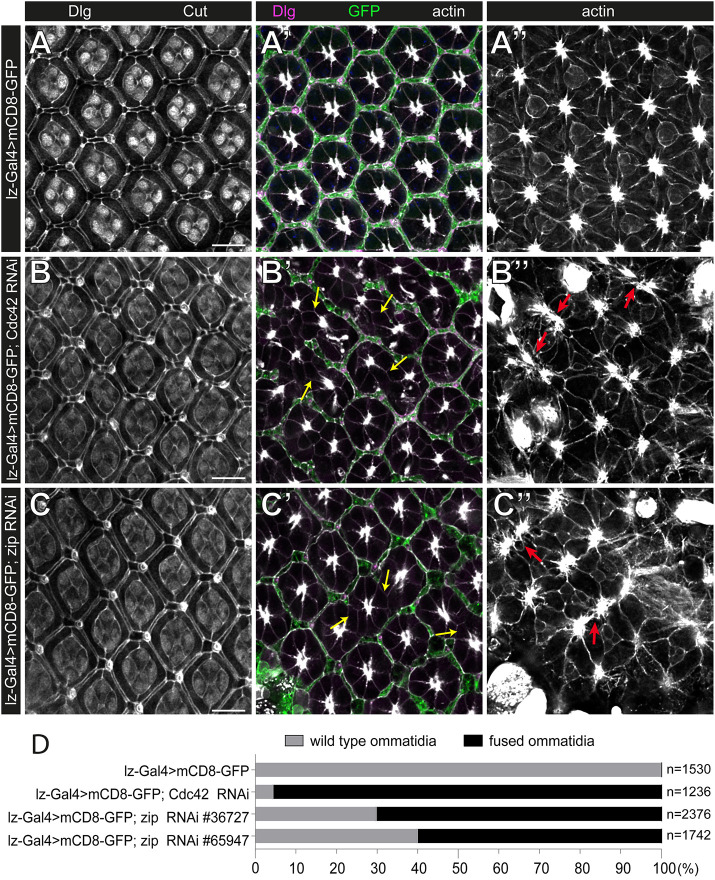
**The RNAi knockdown of *Cdc42* and *zip* results in fused ommatidia, similar to those in *DAAM^Ex4^; frl^59^* double mutants.** (A-C″) Apical (A-C), lateral (A′-C′) and basal (A″-C″) optical sections of control (*lz-Gal4>mCD8-GFP*) (A-A″) and Cdc42 (B-B″) or Zip (C-C″)-depleted eyes stained for Dlg (marking the septate junctions), Cut (marking the cone cell nuclei), GFP (marking the IOCs) and actin. Note that in the control, the GFP-positive IOCs formed a perfectly sealed lattice around the central cells of the ommatidium (A′). RNAi-mediated depletion of Cdc42 had a mild effect on apical patterning of the eye (with minor irregularities due to some shortened horizontal SPCs) (B). However, in the lateral region, the hexagonal IOC lattice was frequently broken (yellow arrows in B′) and the basal eye pattern was also severely impaired, evident by the altered cell shape and axonal exit sites (red arrows in B″). The knockdown of Zip affected the apical hexagonal pattern as the horizontal SPCs or the TPCs appeared to be lost, yet the lattice remained continuous at this level (C). Latero-basal organization of the retina was also impaired, ommatidia fusions were detected at the lateral region (yellow arrows in C′) along with a plethora of retinal floor patterning defects (red arrows in C″), similar to the case of Cdc42 depletion. Scale bars: 10 µm. (D) Quantification of the ommatidia fusion defects in the genotypes indicated. ‘*n*’ indicates the number of ommatidia counted. Data are representative of 12-18 animals per genotype.

Previous work established that activity of the DRF family formins, such as DAAM and FRL, is regulated by Rho GTPases (Chesarone et al., 2010). For example, we found that DAAM is regulated by Rac and Cdc42 but not by Rho1 in the embryonic central nervous system (Matusek et al., 2008), whereas FRL is regulated by Cdc42 in mushroom body neurons of the adult brain (Dollar et al., 2016). Interestingly, Cdc42 and Rho1 have already been linked to retinal morphogenesis by controlling adherens and septate junction organization in the pigment cells (Warner and Longmore, 2009a), but an effect on retinal floor development has not been reported. To determine which GTPase might act together with DAAM and FRL in the pupal eye, we tested loss-of-function mutations of the *Drosophila* Rho GTPases in a series of dominant genetic interaction assays. In these experiments, *Rho1* and the triple Rac mutant for *Rac1*, *Rac2* and *Mtl* did not show a genetic interaction with *DAAM^Ex4^* or *frl^59^* single mutants (Fig. 8), whereas heterozygosity for *cdc42^2^* dominantly enhanced the ommatidium fusion phenotype of *DAAM^Ex4^*/*DAAM^Ex68^* (Fig. 8). Importantly, in the *Cdc42^2^* hemizygous mutant eyes, among other defects, 23% of the ommatidia were involved in a fusion event (Fig. 8). Thus, *Cdc42* appears to display an ommatidium fusion defect on its own, and this was dominantly enhanced by heterozygosity for *frl^59^* (Fig. 8). As further support for this finding, the fusion phenotype was also observed in RNAi-mediated depletion of *Cdc42* in the eye (Fig. 9A-B″,D). Based on these results, we conclude that Cdc42 is likely to control the activity of both formins in the developing eye. Consistent with this possibility, the ChFP-labeled Cdc42 protein (Abreu-Blanco et al., 2012) displayed a strong accumulation along the IOC membranes in the pupal eye (Fig. S8).

The cell shape changes and remodeling of the retinal epithelia are highly dynamic processes, in which a key role for contractile cytoskeletal networks has been well established at the apical AJ and cytoplasmic regions (Blackie et al., 2020; DeAngelis et al., 2020; Del Signore et al., 2018; Johnson et al., 2008). Formins promote the formation of unbranched actin filaments, for e.g. stress fibers, that often participate in contractile acto-myosin complexes (Chesarone et al., 2010; Kühn and Geyer, 2014; Skau and Waterman, 2015). Therefore, we considered the possibility that DAAM and FRL contribute to the assembly of latero-basal acto-myosin systems in the IOCs that are crucial for cell shape and adhesion. If so, we expected that the *DAAM^Ex4^* and *frl^59^* single mutants would show a genetic interaction with *zipper* (*zip*) encoding *Drosophila* non-muscle myosin-II. Indeed, the amorphic *zip^2^* allele exhibited a dominant interaction with *DAAM^Ex4^* and *frl^59^* as judged by the ommatidium fusion phenotype (Fig. 8). Remarkably, depletion of *zip* in the IOCs (by using two independent RNAi lines) phenocopied the retinal floor and lateral defects (Fig. 9C,D) typical for the formin double mutants at 48 h APF. Previous work reported that Zip is highly enriched in the IOC feet (Baumann, 2004) and, here, we found a strong colocalization with FRL and actin (Fig. S9). Collectively, these data suggest that the DAAM- and FRL-dependent cortical actin filaments take part in contractile acto-myosin networks that are crucial in determining the shape and proper adhesion of the IOCs at the latero-basal layers of the eye.

## DISCUSSION

The arthropod compound eye is widely recognized as the most extensively utilized light-sensing organ found in living organisms. Precise sight with such a device critically relies on perfect optical isolation of the individual light-sensing units, which is achieved by a layer of pigment cells, forming thin walls between the central cell clusters of the ommatidia, directly involved in photoreception. In the *Drosophila* eye, three types of pigment-producing cells form a nearly perfect honeycomb lattice, where cell adhesion plays a key role in the stable attachment and maintenance of these delicate cellular structures. Although research over the past decades has successfully unraveled the major mechanisms of the regulation of cell adhesion at the apical junctions, the latero-basal connections remained poorly understood. Here, we describe a previously unreported eye phenotype characterized by defects in the lateral adhesion of the lattice cells. We show that formin-dependent cortical actin assembly is necessary for lateral attachment of the IOCs and we reveal a pivotal formin role in patterning of the retinal floor.

Regulation of the actin cytoskeleton in most cells is orchestrated by several dozens of proteins, some of which possess similar or identical molecular functions. For example, the majority of eukaryotic cells express a number of different formin-type actin assembly factors, required for linear actin cable formation. Despite having similar molecular activities, after careful comparison of their domain composition and amino acid sequences, characteristic differences were also revealed and members of the formin protein family were grouped into several subclasses (Higgs and Peterson, 2005; Pruyne, 2016). Consistent with this diversity, many formin mutants exhibit specific phenotypic effects even if other formins are expressed in the cells affected, although partly redundant functions were also reported in a few cases (Chesarone et al., 2010; Kühn and Geyer, 2014; Dollar et al., 2016). In contrast, DAAM and FRL play completely overlapping roles during *Drosophila* pupal eye development, which is an unusually clear case of functional redundancy. In support of their identical functions in latero-basal eye patterning, they display a largely overlapping protein distribution in the latero-basal region of the IOCs, with a strong cortical membrane accumulation. Curiously, beyond the prominent latero-basal enrichment, both formins are present in the apical region of the eye, albeit lacking a significant overlap in their apical distribution pattern. As neither the *DAAM* and *frl* single mutants nor their double mutants affected apical eye development, these formins may not be required in this region of the eye. Alternatively, the apical role of DAAM and FRL could be redundant with yet another formin. Given that the apical cell area is rich in numerous actin structures and AJs (Del Signore et al., 2018; Johnson et al., 2008), an unrecognized formin requirement is more likely, and we expect that upcoming studies will shed light on this question in the near future.

DAAM and FRL, as members of the DRF formin superfamily, are regulated by an autoinhibitory mechanism that can be relieved upon binding of Rho-family GTPases. Accordingly, our genetic interaction studies fit well with Cdc42 being responsible for DAAM and FRL activation in the latero-basal region of the eye. Remarkably, the genetic depletion of Cdc42 results in highly similar ommatidia fusion phenotypes as the loss-of-function situation for DAAM and FRL, which clearly links Cdc42 to latero-basal eye patterning. In addition, *in vitro* Cdc42-FRL binding has been demonstrated, together with a genetic interaction between *frl* and *cdc42* in the context of ommatidia rotation and axon guidance (Dollar et al., 2016). The Rho GTPases act as key regulators of the cytoskeletal rearrangements, including those involved in cell shape changes and cell adhesion (Etienne-Manneville and Hall, 2002; Fukata and Kaibuchi, 2001), and formins have emerged as their major effector proteins (Kühn and Geyer, 2014). For instance, Dia1 (DIAPH1 in humans) participates in the Rho-dependent regulation of AJs in a human breast cancer epithelial cell line (Carramusa et al., 2007) and in *Drosophila* (Homem and Peifer, 2008), FMNL2 promotes AJ formation downstream of Rac1 in MCF10A cells (Grikscheit et al., 2015), Cdc42 and FMNL3 work together in endothelial cells (Richards et al., 2015) and during wound repair (Rao and Zaidel-Bar, 2016), and Cdc42 and DAAM collaborate to regulate the shape and adhesion of the cardioblast cells during *Drosophila* heart development (Vogler et al., 2014). Whereas most of these studies were focused on the apically located AJs, DAAM1 was shown to be involved in stabilization of lateral cell contacts in a RhoA-dependent manner in the mouse EpH4 cell line (Nishimura et al., 2016). Based on these findings, we propose that in the pupal eye, a Cdc42/DAAM/FRL module is used to determine the shape and ensure stable cell–cell adhesion of the IOCs in the latero-basal layers of the eye. The involvement in latero-basal retina morphogenesis is an unreported function for Cdc42 in *Drosophila* pupal eye development. This role appears to be clearly distinct from its apical function in antagonizing Rho1 activity at the AJs, as Cdc42 promotes the formation and/or maintenance of the cell contacts at the latero-basal IOC membranes, whereas in the apical region, it is a negative regulator of AJ maintenance by promoting DE-cadherin endocytosis (Warner and Longmore, 2009a).

Our rescue experiments and RNAi studies revealed that DAAM and FRL are specifically required in the IOCs but not in other cell types of the eye. As to subcellular specificity, we found a dual role with impairments of the lateral cell contacts, together with alterations in cell shape both at the lateral and basal layers of the retina. A unique feature of the formin loss-of-function phenotype is that the AJs remained largely normal and the IOCs remained attached to the basal lamina; however, in the area between the AJs and the basal lamina, the SPCs often failed to stably attach to the corner cells (TPCs or BCs). In contrast to this, in the most basal, retinal floor area, the feet of the IOCs did succeed in attaching each other and ensured a nearly perfect basal sealing, although they often made unusual contacts never observed in wild-type eyes and exhibited irregular shapes. Thus, if we consider this phenotype along the entire apical-basal axis of the retina, the apical cell contacts look mostly normal, but the lateral connections between the SPCs and the corner cells can be broken almost immediately below the AJ area, all the way down to the endfeet of the IOCs, where they successfully attach to each other again, as well as to that of the underlying basal lamina. In spite of being able to form basal cell connections, the retinal floor pattern is severely compromised in the absence of the formins, as neighbors of the IOCs, and the shape and arrangement of their feet significantly differ from those in wild type. Because the IOC feet can still attach to the basal lamina and, to some extent, each other even in the absence of proper lateral connections, it remains an open question whether the lateral and basal patterning defects are independent of each other or the basal defects are the indirect consequences of the lateral defects (or vice versa). As the wild-type retinal floor pattern appears highly complex, we favor the possibility that DAAM and FRL contribute to the formation of an actin network specifically dedicated to retinal floor development, which is distinct from the more apically located actin cables involved in lateral attachment of the cells.

Consistent with the well-known formin functions in actin regulation, we detected reduced cortical actin levels in the latero-basal areas of the IOCs and also at the level of the AJs. Because the formin-dependent ommatidia fusion phenotype is sensitive to non-muscle myosin levels, and the knockdown of Zip also results in fused ommatidia, the cortical actin subpopulation regulated by DAAM and FRL is likely to be contractile, as described for other actin networks involved in cell adhesion (Harris and Tepass, 2010; Mege and Ishiyama, 2017). On this ground, we suggest that the primary function of DAAM and FRL is linked to the establishment and maintenance of proper cell contacts between the IOCs, and the profound effect on cell shape can be explained as an indirect consequence of losing cellular connections. Nonetheless, the IOCs exhibit a peculiar cell shape necessary to form very thin walls between and underneath the central cells of the ommatidial clusters. Various cytoskeletal elements, primarily actin filaments, are expected to play a role in the formation of such unique shapes, and, accordingly, several cortical and cytoplasmic actin populations are implicated in IOC development at the apical junctional area (DeAngelis et al., 2020; Del Signore et al., 2018). Our data clearly point toward the importance of cortical actin filaments in the latero-basal region; however, we did not detect the presence of any obvious cytoplasmic actin networks. Whereas properly organized and attached cortical actin structures might be sufficient to form and maintain the thin walls formed by the SPCs in the lateral region of the eye, the formation and maintenance of the flower petal pattern of the IOC feet (composed of the combination of concave and convex cell borders) is more difficult to reconcile with exclusively cortical actin-dependent mechanisms that do not involve cytoplasmic actin populations. Therefore, it appears more likely that technical limitations prevent the detection of the cytoplasmic actin populations that are also at work in shaping the retinal floor. Alternatively, the regulation of cytoplasmic tension might also be a key factor in the formation of the IOC feet, and differential regulation of the resistance of the cortical actin in the SPCs versus TPCs and BCs might be sufficient and necessary to dictate the shape of their feet. Although further investigations will be required to clarify these questions, we trust that the analysis of these previously unreported formin-dependent eye phenotypes will be highly beneficial to better understand the three-dimensional aspects of pupal eye morphogenesis.

## MATERIALS AND METHODS

### *Drosophila* stocks

*Drosophila melanogaster* stocks were raised on standard cornmeal-yeast-agar medium at 25°C. The following mutant strains were used: *w^1118^* (BL#3605), *54C-Gal4* (BL#27328), *UAS-LifeAct::GFP* (BL#58718), *UAS-α-Catenin::GFP* (BL#58787), *RhoA^72F^* (BL#7326), *Rac1^J11^*, *Rac2Δ*, *MtlΔ* (BL#6678), *Cdc42^2^* (BL#9105), *zip^2^* (BL#8739), *UAS-mCD8::GFP* (from BL#5134), *FRL RNAi* (BL#32447), *Cdc42 RNAi* (BL#37477), *zip RNAi* lines (BL#36727 and BL#65947), *sqh-ChFP::Cdc42* (BL#42236) and *DAAM RNAi* (BL#39058), all from the Bloomington *Drosophila* Stock Center; *DAAM^Ex68^*, used as a *DAAM* null allele (Matusek et al., 2006); *DAAM PD RNAi* [the same construct as in Chougule et al. (2020), inserted in the attP40 site]; *GMR-Gal4* and *lz-Gal4>mCD8-GFP* (kind gifts from Jessica E. Treisman, New York University); *UAS-DAAM PB* [the same DAAM sequence as in (Matusek et al., 2006) was cloned into pUAST-attB and integrated into an attP40 landing chromosome]; *DAAM^Ex4^* and *UAS-FLDAAM I732A* (Gombos et al., 2015); *UAS-FRL* and *UAS-FRL I773A* (see below); and *frl^59^* (Dehapiot et al., 2020). The *Drosophila* database Flybase (release FB2022_06) (Gramates et al., 2022) was used to retrieve genetic data.

### *Drosophila* genetics

The *DAAM^Ex4^>GMR-Gal4* and *DAAM RNAi>frl^59^* lines were generated by standard genetic recombination techniques. *UAS-FRL* was constructed with standard molecular cloning techniques, and subsequently used as template to create UAS-FRL I773A with PCR mutagenesis. The 5′-CGTCGCAAGCTGGGTATGCCC-3′ and 5′-GGACGCTGCAATGTTTCTTAACCTCGTGTGC-3′ primers were used to mutate Ile773 to alanine in *UAS-FRL I773A*.

### Immunohistochemistry

Pupae were collected at 0 h APF and maintained at 25°C until dissection. They were dissected at 48 h APF in ice-cold PBS, fixed in 4% paraformaldehyde (diluted in PBS) at room temperature for 20 min. After fixation, the samples were washed in PBS containing 0.1% Triton X-100 (PBST) three times for 20 min, and blocked in PBST with 0.2% BSA (PBS-BT) for 2 h. Primary and secondary antibodies were diluted in PBS-BT and incubated overnight at 4°C. The samples were mounted in PBS:glycerin (1:4) or in a ProLong Gold reagent (P36930, Thermo Fisher Scientific).

The primary antibodies used in this study were: rat anti-FRL (1:1000; Toth et al., 2022), rabbit anti-dDAAM (R4) (1:500; Gombos et al., 2015), rabbit anti-Zip (1:200; Chougule et al., 2016), chicken anti-GFP (1:1000, ab13970, Abcam), mouse anti-Dlg [1:100, 4F3, Developmental Studies Hybridoma Bank (DSHB)], mouse anti-chaoptin (1:50, 24B10, DSHB), mouse anti-Armadillo (1:500, N27A1, DSHB), mouse anti α-catenin (1:100, DCAT-1, DSHB), mouse anti-Cut (1:500, 2B10, DSHB), rat anti-DE-cadherin (1:100, DCad2, DSHB), rat anti-N-cadherin (1:10, DN-Ex#8, DSHB), mouse anti-Talin (1:10, E16B, DSHB) and mouse anti-Mys (1:100, CF.6G11, DSHB).

As secondary antibodies, we used the appropriate Alexa Fluor 488- or Alexa Fluor 647-coupled antibodies (1:600; anti-mouse Alexa 488, A-11001; anti-mouse Alexa 647, A-21235; anti-chicken Alexa 488, A-11039; anti-rabbit Alexa 647, A-21245; anti-rat Alexa 488, A-11006; anti-rat Alexa 647, A-21247; Thermo Fisher Scientific). Actin was labeled with either Phalloidin-Alexa Fluor 488 or -Alexa Fluor 546 (1:50; Phalloidin-Alexa 488, A12379; Phalloidin-Alexa 546, A22283; Thermo Fisher Scientific).

### Retina cell number counting

To count the ommatidial cell numbers in wild type and formin double mutants, pupae at 48 h APF were dissected and stained for DE-cadherin, allowing the counting of CCs, PPCs, SPCs, TPCs and BCs. RCs were counted based on chaoptin staining. At least seven to ten retinas, with at least 100 ommatidia, were counted for both genotypes. ImageJ/Fiji (Schindelin et al., 2012) and Microsoft Excel 2016 were used to quantify the cell types.

### Live imaging, image analysis and quantification

Live imaging was performed as described previously (Larson et al., 2008). Every video was acquired at 25°C. We filmed *z*-series of 50 planes separated by 0.6 µm and acquired at a frequency depending on the number of examined pupal eyes. Images were restored using Huygens Professional (Scientific Volume Imaging B.V., Hilversum, The Netherlands) and ImageJ/Fiji software. ImageJ/Fiji and Microsoft Excel 2016 was used to quantify the different parameters of the eye.

To trace the IOCs across the entire apical-basal axis of the eye, we expressed *mCD8-GFP* with *54C-Gal4* to label the SPCs and TPCs. In addition, Dlg staining was used to mark the septate junctions, anti-Cut to mark nuclei of the CCs and the BC complex, and phalloidin to label F-actin. Confocal *z*-series were taken with a 0.14 µm slice distance, resulting in about 90-100 optical sections for every sample. The shapes of the IOCs (SPCs, TPCs and BCs) were contoured and color-coded manually on every optical section, allowing us to follow the cell shape from the most apical to the basal layers of the retina.

To assess the level of colocalization between DAAM and FRL, we determined Pearson's correlation coefficient. For this analysis, images were captured in accordance with the Nyquist rate, using the full dynamic range of the detectors. Prior to quantification, images underwent restoration using Huygens Professional software. Pearson's correlation coefficient was determined using Fiji's Coloc2 plugin. For the measurements restricted to the IOCs, we used manually outlined masks.

For the comparison and quantification of actin intensity in wild-type and formin double-mutant eyes, staining with Phalloidin-Alexa Fluor 488 was carried out in the same Eppendorf tube to ensure the exact same conditions (the wild-type and mutant eyes were subsequently sorted based on their phenotype). Actin intensity was measured using ImageJ/Fiji at the cortical membrane in the apical and lateral region of the eye (on single optical sections 3 and 6 µm below the apical surface, respectively) along a manually drawn line following the cell cortex. Five to ten eyes were analyzed per sample, and 60-70 cells were measured on each image depicting an apical or lateral layer. The values were normalized to the average of the background intensities recorded at several areas of the image.

For quantification of the ommatidia fusion phenotype, eyes were stained with anti-chaoptin (1:50, 24B10) at 48 h APF. On the confocal images, fused and wild-type ommatidia were counted manually using ImageJ/Fiji. An average of ten to 16 eyes (from at least two independent stainings) were counted per genotype.

All imaging was done with a Zeiss LSM800 confocal microscope, using 63×/NA 1.4 oil or 40×/NA 1.3 objectives.

### Statistics and figures

Statistical analysis was carried out using Prism 8 (GraphPad Software, San Diego, CA, USA). The D'Agostino–Pearson omnibus test was used to assess the normality of the data. Significance levels were calculated with Mann–Whitney test (****P≤0.0001). Figures and drawings were created in Illustrator CS6 (Adobe). The graphical 3D reconstructions were created with Blender 3.5. (Stichting Blender Foundation, Amsterdam).

## Supplementary Material

10.1242/develop.201713_sup1 Supplementary information
