# Supplementary material for: FRL and DAAM are required for lateral adhesion of interommatidial cells and patterning of the retinal floor
Source: Development. 2023 Nov 24;150(22):dev201713. doi: 10.1242/dev.201713 (PMC10690107; doi:10.1242/dev.201713)
Supplement: Supplementary information [file develop-150-201713-s1.pdf]

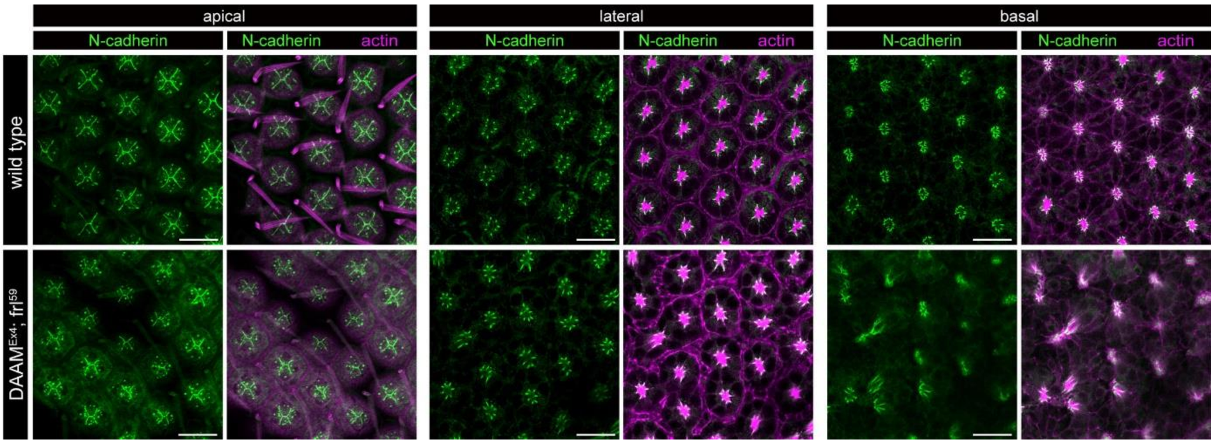

Fig. S1. The localization of N-cadherin remains unaltered in the formin mutant eyes. Confocal Z-sections of a wild type and a *DAAM*^{Ex4}; *frl*^{59} double mutant eye at 48 h APF stained for N-cadherin (in green) and actin (in magenta). Note that N-cadherin is expressed in the AJs of the CCs (visible in the apical section), and at the AJs of the photoreceptor cells (visible in lateral and basal sections, and running perpendicular to the plane of these sections in the wild type eye). The N-cadherin pattern of the formin mutants looks essentially identical to that of wild type in all sections. Note that the seeming difference in the basal section is caused by altered orientation of some of the rhabdomeres and AJs as compared to the normal optical axis of the ommatidia. Scale bars, 10µm.

Development • Supplementary information

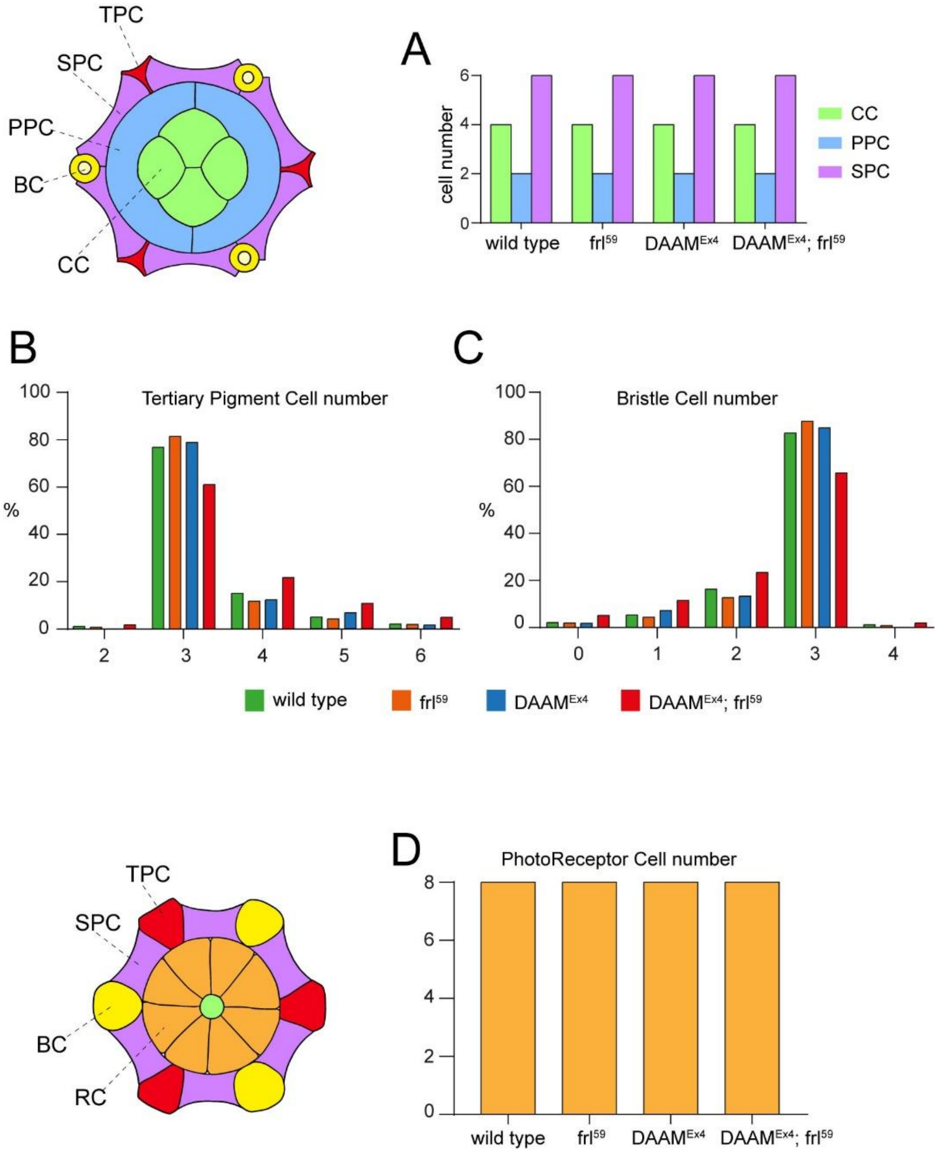

Fig. S2. Quantification of cells number in the wild type and formin mutant eyes. The number of the 4 cone cells (CC), 2 primary and 6 secondary pigment cells (PPCs and SPCs) remains unchanged in $DAAM^{Ex4}$ and frl^{59} single mutants, and also in the double mutants as compared to wild type (A). The number of 3 tertiary pigment cells (TPCs) (B) is sometimes increased to 4 or 5 in the formin double mutants, whereas number of the 3 bristle cells (BC) (C) is reduced to 0-2 in a small portion of the $DAAM^{Ex4}$; frl^{59} double mutant eyes. The number of photoreceptor cells (RCs) is the same in wild-type and mutant animals (D).

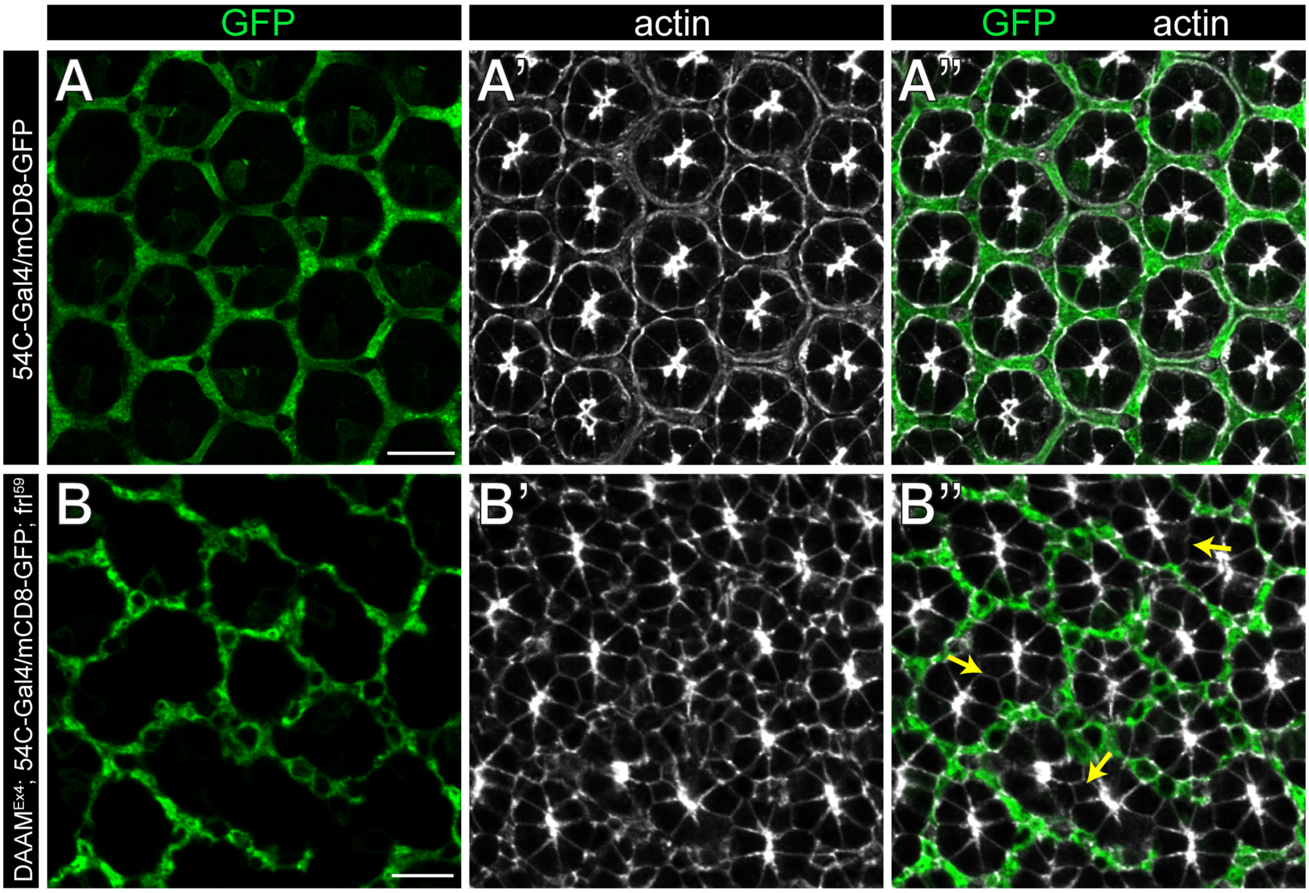

Fig. S3. Representation of the ommatidial fusions with expression of mCD8-GFP in the IOCs. Optical Z-section of the lateral eye region of control (*54C-Gal4/UAS-mCD8-GFP*) (A-A") and formin double mutant eyes (also marked by *54C-Gal4/UAS-mCD8-GFP*) (B-B") stained for GFP and actin. Note that GFP is present in the SPCs and TPCs, forming thin walls between the ommatidia in the wild type eye (A, A"). As compared to this, the SPCs and TPCs often acquire an abnormal shape, and often fail to connect each other (B, B") (yellow arrows in B"), resulting in a broken lattice in the double mutant eye (B-B"). Actin is used to label the R cells and the bristle cell complex as well (A', A", B', B"). Scale bars, 10µm.

Development • Supplementary information

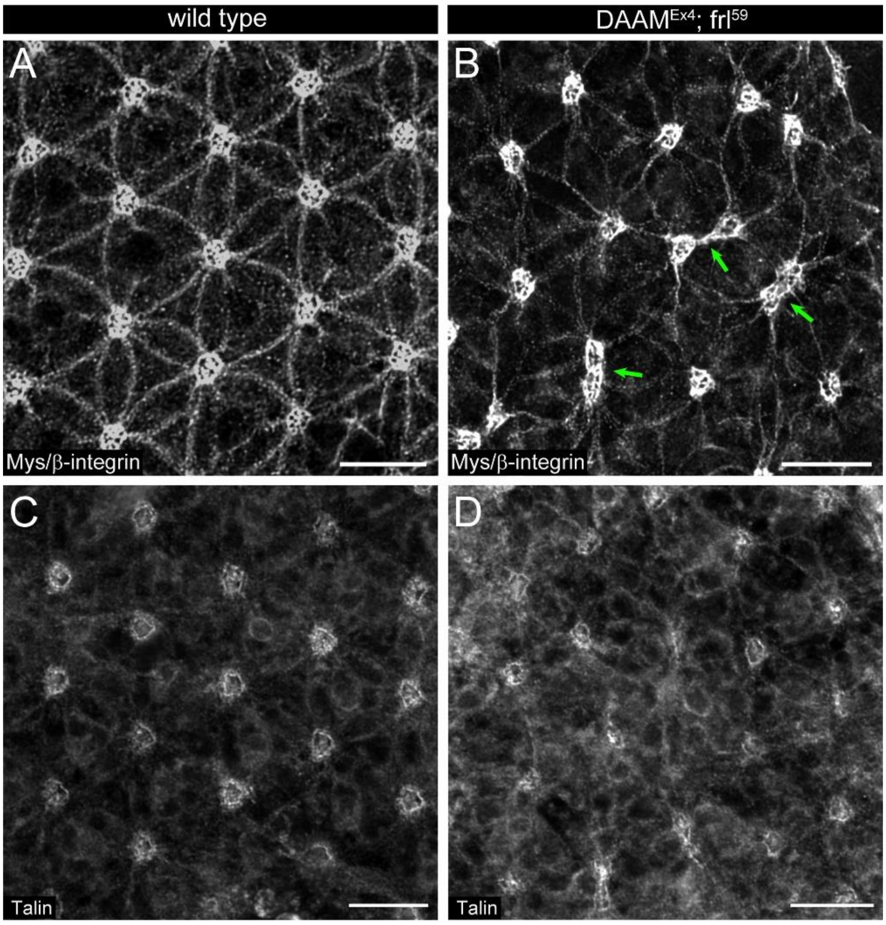

Fig. S4. The basal focal adhesion sites are present in the formin double mutant eyes. Confocal immunofluorescent images of pupal eyes at 48 h APF, stained for Mys/β-integrin (A, B) and Talin (C, D) to mark the grommets and the basal focal adhesion sites. In a wild type eye (A) Mys reveals the flower petal pattern of the IOC feet. The basal focal adhesion sites are also present in a *DAAM^{Ex4}*; *frl^{59}* double mutant eye (B), but uneven spacing and occasional fusion of the grommets (green arrows) is obvious, as well as the irregular shape and position of the lattice cell feet. (C) Talin is also enriched at the grommets, at it is present along the IOC cell borders as well as at several other more diffused site in the basal region. (D) As expected, the Talin pattern in the formin double mutants is less regular, yet the grommets are clearly recognizable, and there is no obvious change in the expression level. Scale bars, 10 µm.

Development • Supplementary information

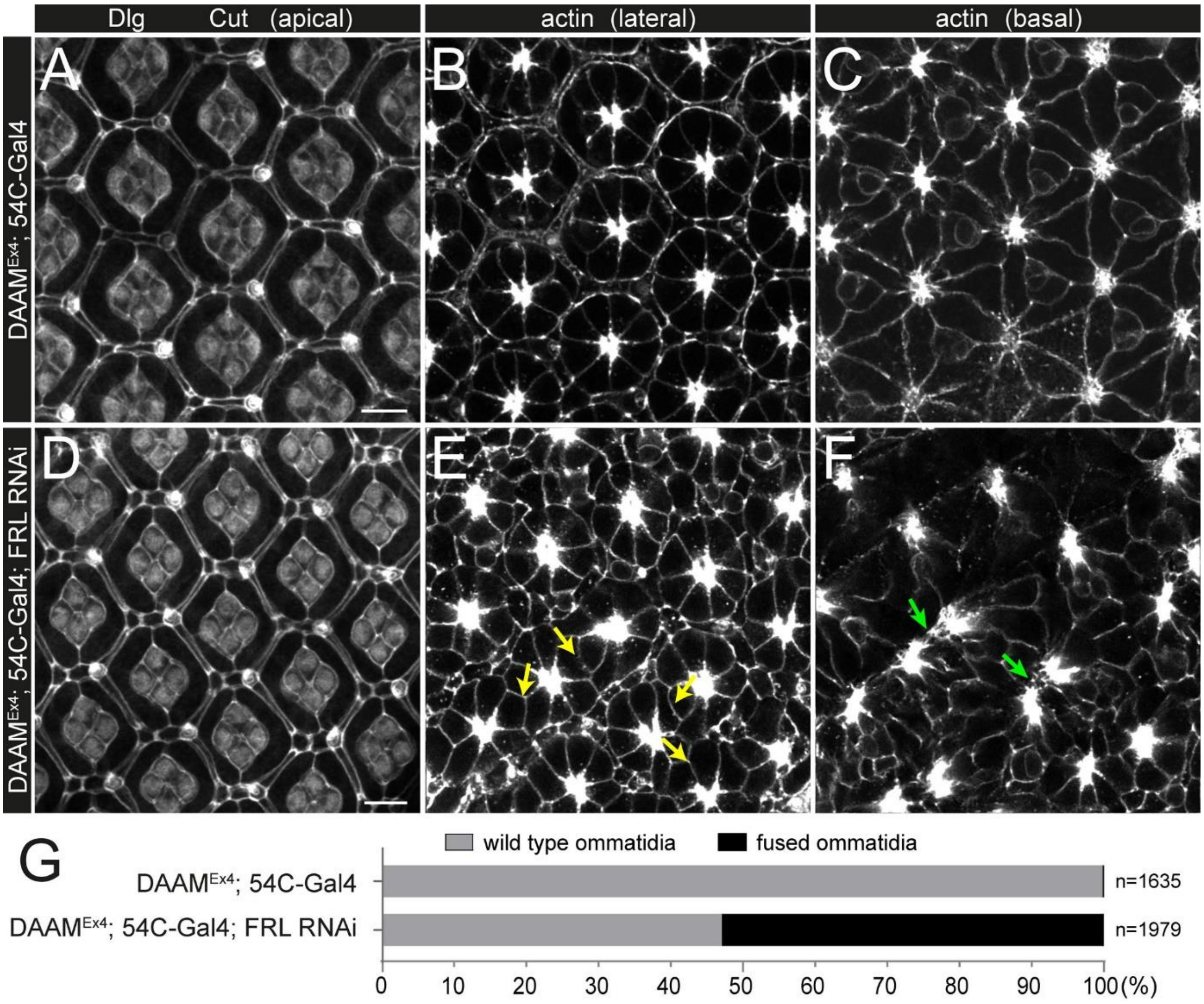

Fig. S5. IOC specific silencing of *frl* in a *DAAM*^{Ex4} mutant background results in eye phenotypes typical for the double formin mutants. Confocal Z-sections of pupal eyes from the *DAAM*^{Ex4}; *54C-Gal4*/+ (A-C) and *DAAM*^{Ex4}; *54C-Gal4*/+; *FRL*^{RNAi}/+ (D-F) mutant combinations; eyes were stained for Dlg (marking the SJs), Cut (marking the cone cell nuclei) and actin. Eyes from the *DAAM*^{Ex4}; *54C-Gal4*/+ controls exhibit a wild type morphology at the apical (A), lateral (B) and basal (C) layers of the eye. IOC specific silencing of FRL in the *DAAM*^{Ex4}; *54C-Gal4*/+ background results in slightly impaired horizontal SPCs (D), and in ommatidia fusions (E, yellow arrows) and basal patterning defects, i.e. cell shape changes and irregularly spaced axonal exit sites (F, green arrows). (G) Quantification of the ommatidia fusion phenotype shown in A-F. “n” indicates the number of ommatidia counted. Scale bars, 10µm.

Development • Supplementary information

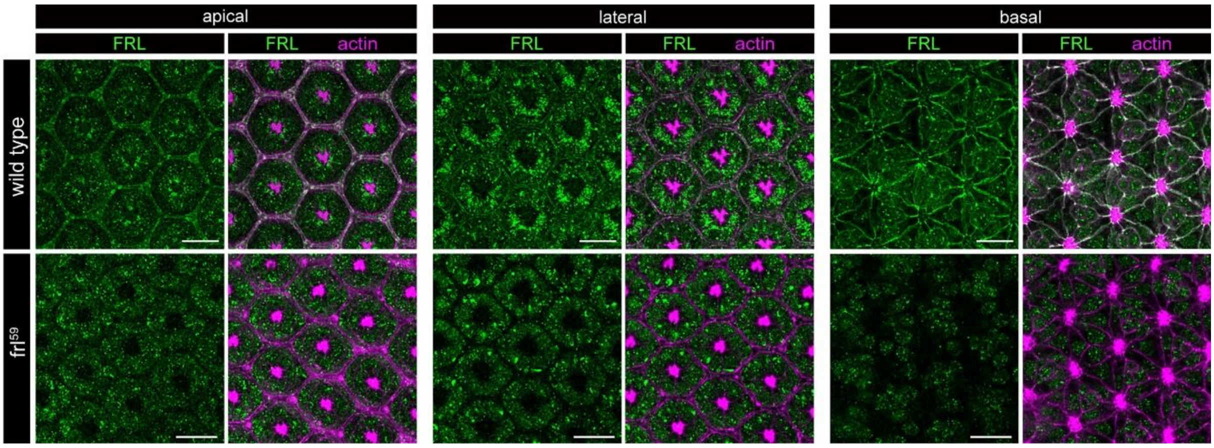

Fig. S6. Immunhistochemical specificity of the FRL antibody. To assess the specificity of the anti-FRL serum in pupal eye staining, we compared the staining pattern in a wild type and an *frl*^{59} mutant eye. FRL is shown in green, actin is labeled in magenta. In the apical region of a wild type eye FRL is present in the cortical membrane of the IOCs, and a largely dotty pattern is visible in the central cells of the ommatidia. As compared to this, in the formin (null) mutant eye the IOC specific staining is not visible, however, the partly dotty, partly diffuse pattern in the central cells remains detectable. In the lateral layer of a wild type eye FRL accumulates along the cortical membrane of the IOCs, and a dotty (vesicular looking) pattern is obvious in the RCs. Because in the mutant situation the IOC staining is not visible, while the RCs staining remains clearly detectable, we conclude that the RC staining is very likely to represent a non-specific background. In basal region of the eye FRL is mostly detected along the cortical membrane of the IOCs, together with some cytoplasmic dots. As the latter (partly dotty, partly diffused) pattern is present in the mutant, while the IOC membrane staining is absent, these observations confirm that the anti-FRL specifically labels the IOC cortical membranes, yet a weak non-specific signal can also be detected with it. Scale bars, 10µm.

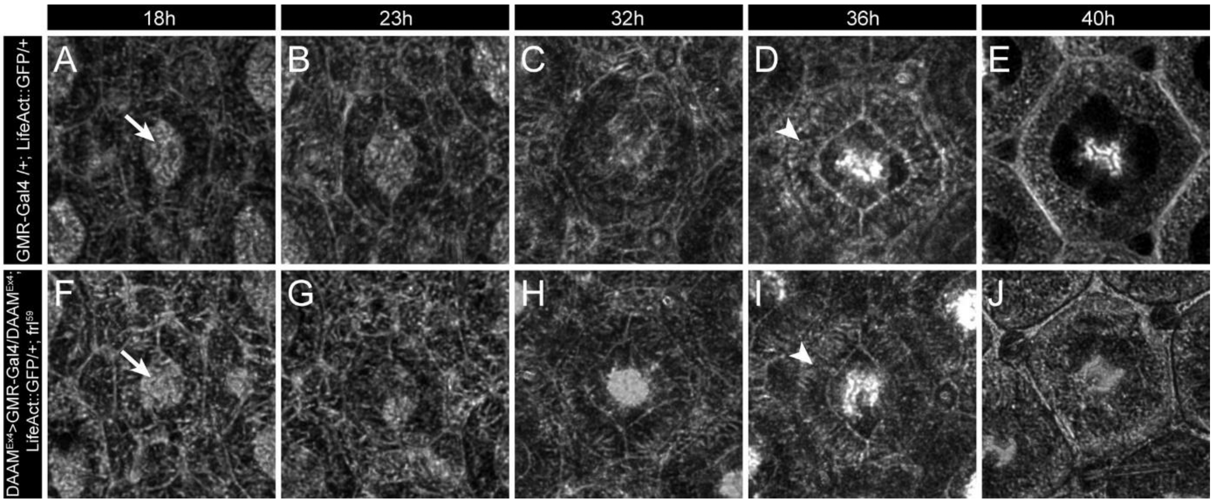

Fig. S7. The analysis of apical F-actin organization during pupal eye development with live-imaging microscopy. Snapshots of live-imaging movies of a control (A-E) and a *DAAM*^{Ex4}; *frl*^{59} double mutant eye (F-J) expressing LifeAct::GFP in all retinal cells (with GMR-Gal4) at five developmental time points between 18-40 h APF. A single ommatidial cluster is shown in both cases. F-actin is accumulated at the AJs and it forms an intricate mesh, particularly evident in the cone cells (arrows in A, F) and the PPCs (arrowheads in D, I). No clear differences were detected between the wild-type and mutant eyes.

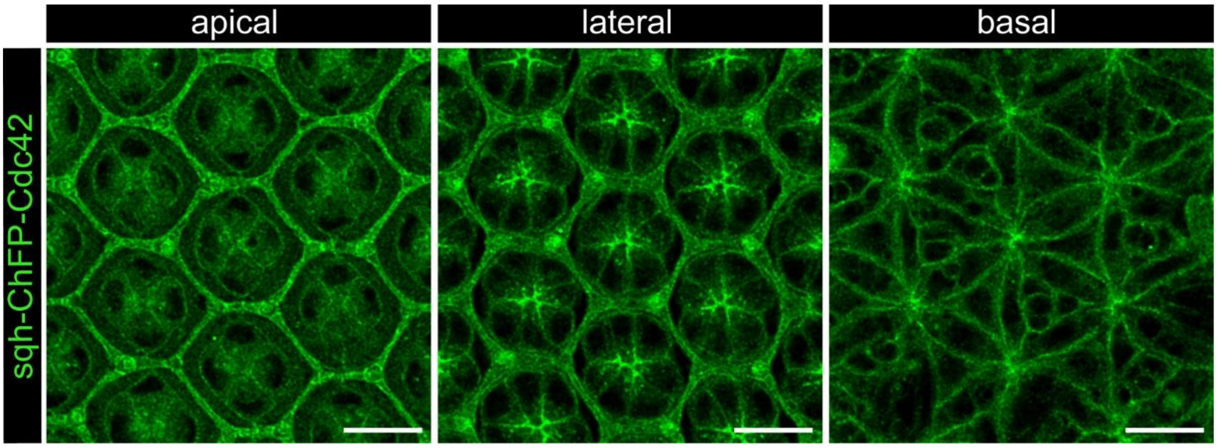

Fig. S8. The distribution pattern of ChFP::Cdc42 in the pupal eye. To examine the subcellular localization of Cdc42 in the 48 h APF pupal eye we used a ChFP labelled Cdc42 expressed under the control of an *sqh* promoter. Direct visualization of the fluorescently labelled Cdc42 protein revealed a strong accumulation at the cellular junctions in the apical, lateral and basal sections as well. The enrichment at the cell-cell contacts is particularly evident in the IOCs (in all layers of the eye), that also appear to exhibit some cytoplasmic Cdc42 punctae in the apical and lateral sections. Scale bars, 10µm.

Development • Supplementary information

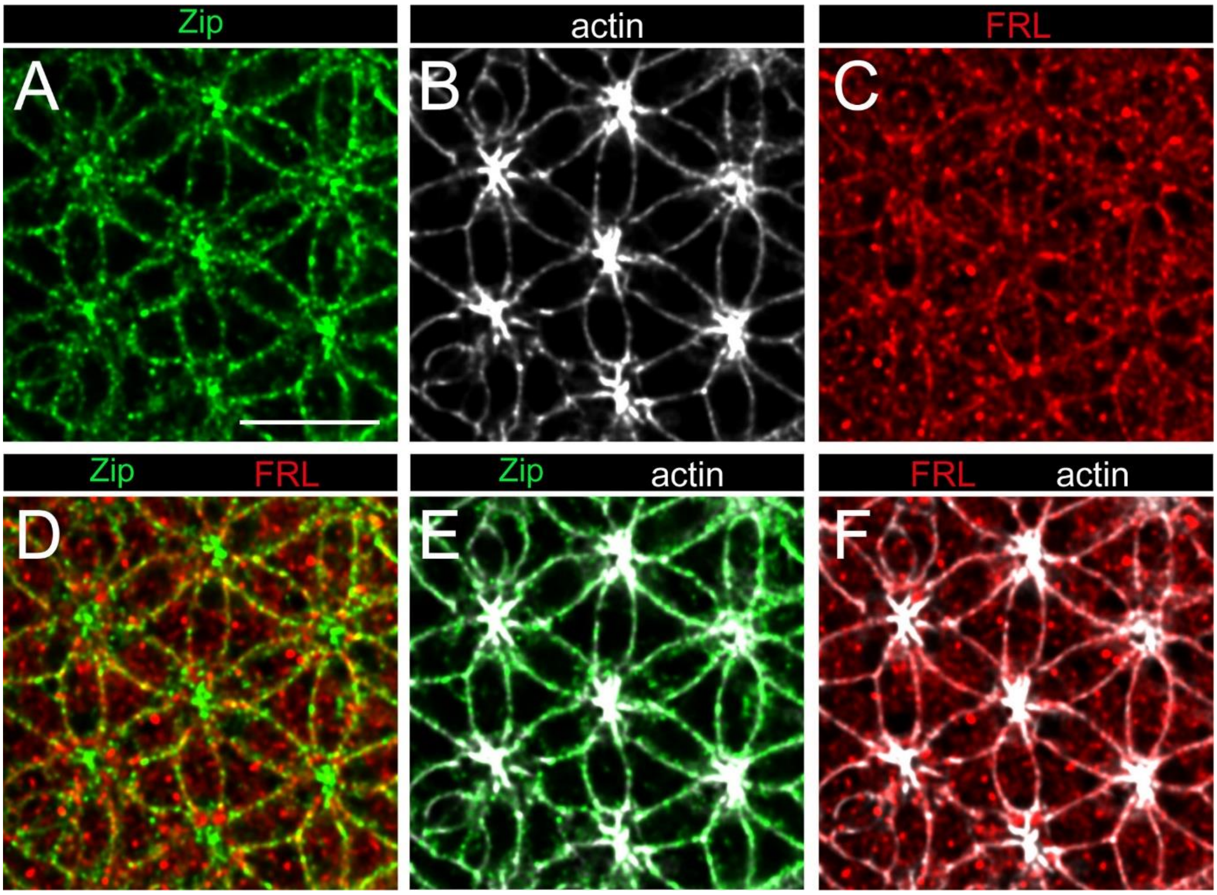

Fig. S9. Zip strongly colocalizes with FRL and actin in the IOC feet. (A-F) Confocal image of a wild type pupal eye at 48 h APF stained for Zip, FRL and actin. Zip shows a strong cortical membrane accumulation at the IOC feet (A) where it colocalizes with actin (B, E) and FRL (C, D), actin and FRL exhibiting a colocalization as well (F). Scale bar, 10µm.

Development • Supplementary information
