# Supplementary material for: FRL and DAAM are required for lateral adhesion of interommatidial cells and patterning of the retinal floor
Source: Development. 2023 Nov 24;150(22):dev201713. doi: 10.1242/dev.201713 (PMC10690107; doi:10.1242/dev.201713)
Supplement: Supplementary file 1 [file develop-150-201713_review_history.pdf]

FRL and DAAM are required for lateral adhesion of interommatidial cells and patterning of the retinal floor

Gabriella Gazsó-Gerhát, Rita Gombos, Krisztina Tóth, Péter Kaltenecker, Szilárd Szikora, Judit Bíró, Enikő Csapó, Zoltán Asztalos and József Mihály
DOI: 10.1242/dev.201713

Editor: Thomas Lecuit

Review timeline

Original submission:	17 February 2023
Editorial decision:	17 April 2023
First revision received:	17 August 2023
Editorial decision:	6 October 2023
Second revision received:	9 October 2023
Accepted:	17 October 2023

Original submission

First decision letter

MS ID#: DEVELOP/2023/201713

MS TITLE: FRL and DAAM are required for lateral adhesion of the interommatidial cells and patterning of the retinal floor

AUTHORS: Gabriella Gazso-Gerhat, Rita Gombos, Krisztina Toth, Peter Kaltenecker, Szilard Szikora, and Jozsef Mihaly

I sincerely apologise for the delay. I have now received all the referees reports on the above manuscript, and have reached a decision. The referees' comments are appended below, or you can access them online: please go to BenchPress and click on the 'Manuscripts with Decisions' queue in the Author Area.

The overall evaluation is positive and we would like to publish a revised manuscript in Development, provided that the referees' comments can be satisfactorily addressed. Please attend to all of the reviewers' comments in your revised manuscript and detail them in your point-by-point response. If you do not agree with any of their criticisms or suggestions explain clearly why this is so. If it would be helpful, you are welcome to contact us to discuss your revision in greater detail. Please send us a point-by-point response indicating your plans for addressing the referee’s comments, and we will look over this and provide further guidance.

Reviewer 1

Advance summary and potential significance to field

Gazsó-Gerhát, Gombos and colleagues identify a requirement of two formins, FRL and DAAM, for the basolateral organization of Drosophila interommatidial cells.
The combined requirement is confirmed with loss-of-function alleles and RNAi constructs. Notably, apicolateral cell-cell junctions and cell architecture are minimally affected. The basolateral defects are rescued by cell-type-specific expression of wild type FRL or DAAM constructs, but not by constructs containing mutations known to disrupt actin binding and that often display dominant

© 2023. Published by The Company of Biologists under the terms of the Creative Commons Attribution License (https://creativecommons.org/licenses/by/4.0/).

negative effects on their own. Moreover, basolateral levels of cortical actin are reduced in the mutants. Consistent with their basolateral requirement, both FRL and DAAM display greater cortical enrichment laterally and basally versus apically. Genetic interactions, phenocopies, and partial localization data implicate involvement of Cdc42 and Zipper in the FRL/DAAM role. Overall, this paper describes a carefully conducted study, and is of interest for identifying molecular bases of basolateral structure of Drosophila eye cells, a prominent model system which has mainly been studied in terms of apicolateral organization.

Comments for the author

A number of revisions would improve the paper.

1. Substantial quantifications are included. As much as possible, the authors should indicate the number of animals quantified. This inclusion is especially important when applying statistical tests, which should be done with N equal to the number of animals rather than N equal to the number of ommatidia or cortical ROIs, a concern relevant to the basolateral F-actin measurements in Fig 6E.

2. To test if the effect on basolateral F-actin is specific, levels of apicolateral F-actin should also be quantitatively compared between control and the double mutants.

3. A underdeveloped aspect of the paper is the effect of the double mutants on lateral cell-cell adhesion. Is local cell-cell adhesion affected or unaffected in addition to the dramatic cell shape changes? Local effects on adhesion could be examined by immunostaining of candidate cell adhesion molecules and/or by TEM of lateral cell-cell contacts.

4. The side views in Figure S3 G-I are informative and would be good to include in a main figure.

5. It would be good to confirm that the double mutant eyes are defective in light transmission, using the deep pseudopupil (DPP) assay.

6. The specificity of the in situ immuno-staining with Frl and DAAM antibodies should be confirmed by staining eyes with experimental depletion of Frl or DAAM or by referencing papers where this test may have been done. Testing specificity with western blots is insufficient.

7. Data showing Cdc42 localization should be added or referenced.

<u>Reviewer 2</u>

Advance summary and potential significance to field

In this study, Gazso-Gerhat and colleagues, examine the role the Formins FRL and DAAM, play in Drosophila retina morphogenesis. They combine genetic experiments and imaging of staged retinas to characterize the function of these two F-actin effectors in apical and basal surface organization.

The Drosophila retina is an ideal model system to study the pathways that govern epithelial surface patterning and morphogenesis, as cells adopt stereotypical shapes during development.
The main finding of this study is that FRL and DAAM act redundantly in retinal morphogenesis. These Formins are required for normal organization of the basal tissue surface, and they appear to mostly function in the interommatidial cells, which make up this surface – this is demonstrated with a very nice rescue experiment employing the 54C-Gal4 strain, which is expressed in these cells, but not in the photoreceptors, primary pigment, and cone cells. Overall, the paper is interesting because not much is known of the pathways that control the 3D shape of epithelial cells. The experiments are well executed, and the quality of the data is excellent.

However, there are a few issues with the paper, which relate to how the authors interpret some of their experiments. I think they are overreaching in their conclusions, which leads to proposing a model that is not well supported by the data. Considering the phenotypes they observe, and pattern of expression they document for FRL and DAAM, the authors conclude that these factors are required for lateral adhesion between the interommatidial cells and also play a direct role in setting the basal morphology of these cells. I am not convinced this is the case, based on the data

© 2023. Published by The Company of Biologists under the terms of the Creative Commons Attribution License (https://creativecommons.org/licenses/by/4.0/).

they present in the paper. What is the evidence for lateral adhesion? Figure 3 shows that all interommatidial are present at the apical surface of the tissue. More basally, some are no longer detected, and accordingly, when reaching the basal surface many are missing.
This suggest a failure for some of these cells to reach the basal surface – elongation failure? If some interommatidial are not contributing to the basal surface, then this should lead to significant defects in basal surface organization. This does not necessarily involve a direct role for these Formins in regulation the basal cell cortex. Instead, it could be a consequence of defect in cell packing, basally. I think more work is needed to ensure the main conclusion presented in the paper is correct.

Comments for the author

- A quantitative analysis of the expression patterns for Frl and DAAM, where a co-localization study would be advantageous. This would make it easier to understand where these co-localize and where they do not. FRL seems quite vesicular in the photoreceptors and interommatidial cells?
- Figure 3: the apical membrane of the photoreceptors seems affected. The authors should describe this aspect of the phenotype, if only briefly. It fits with the expression pattern of DAAM, which seems localised in these cells, enriched apically.
- To my eyes, it is not possible to separate the basal membrane of the photoreceptors and the membrane of the interommatidial cells– these plasma membranes are apposed. This makes it very difficult to ascertain whether the staining for Frl, DAAM (Fig.5) and F-actin (Fig.6) belong to the photoreceptors or to the interommatidial cells. This is especially problematic since both Formins are expressed in the photoreceptors.
Perhaps the authors could make use of the 54CGal4 driver to inhibit frl/DAMM in the interommatidial cells (e.g. DAAM-/-; 54C>frl RNAi) and quantify this genotype?
- Line 90: Longley & Ready show a requirement for integrin in basal contraction, not in patterning. The authors should modify their statement accordingly.
- Line 163: we expressed …/… in the developing IOCs by 54C-Gal4: Figure 3 does not show this.
- Line 194: " misoriented rhabdomeres": what do the authors mean by this?
- Scale bars are missing in some figures, for example Fig.5.
- Line 305: "we conclude that cdc42 controls the activity of both Formins: This conclusion is not very solid as it is based on the finding that the phenotypes are similar and that they interact genetically. To back up this conclusion, the authors need to show evidence that these Forming act downstream of cdc42.. For example they could express Frl or DAAM in cdc42 retinas and assess whether this rescues the cdc42 phenotype. They could also quantify the expression of Frl and DAAM in the cdc42 retinas, of when using the cdc42 RNAi.
- They use many RNAi lines for their work: how here these RNAi lines validated to ensure they to not come with off-target effects?

<u>Reviewer 3</u>

Advance summary and potential significance to field

This manuscript by Gazsó-Gerhát and colleagues examines the potential redundant function of two formins, FRL and DAAM, in the Drosophila retina. They demonstrate that single mutants in either gene result in near wild-type eyes, but disruption of both simultaneously leads to deep defects in pigment cell separation of photoreceptor clusters (and other seemingly more minor defects). The authors argue that the effects of double disruption in apical layers is less significant than those in more lateral and basal regions. Although the exact mechanism of action is somewhat unclear, it appears that basal focal adhesions are disorganized, and phenotypes are exacerbated when myosin (zipper) is also disrupted. The authors suggest that contractile actin filaments (which do not appear to be be imageable in this system) promote adhesive function between interommatidial cells/pigment cells and/or the function of the basal focal adhesions. The manuscript is well-written, with a nice Introduction, and the Results are (largely) appropriately described and interpreted. The genetic analysis appears well-done, and the rescues are very nice and convincing. The quantitation appears appropriate, though with little in the way of tests for statistical significance. It is a farily straightforward story that builds on an earlier paper involving similar formin function during ommatidial rotation and neuron outgrowth (Dollar et al., 2016). A few more detailed comments are below:

© 2023. Published by The Company of Biologists under the terms of the Creative Commons Attribution License (https://creativecommons.org/licenses/by/4.0/).

Comments for the author

1) As a potential approach to better get mechanism, what do the cadherins look like in these backgrounds?
This would seem to allow for a better argument for a FA vs cadherin mechanism.

2) Similarly, could overexpression of Zipper, Sqh, or activated Rho be done to see if FAs are weakened, or if particular regions of retinal organization appear to be differentially affected?

3) Line 213, “The ommatidium fusion phenotype and the retinal floor defects observed in the DAAMEx4; frl59 mutants indicated a formin requirement in the IOCs”. This does not seem accurate (“IN the IOCs”); at this point in the manuscript it could still be through non-autonomous effects.

4) Line 305-306 – it can’t be formally concluded that Cdc42 controls formin function based off of these experiments (absent Frl or DAAM staining or epistasis), though I'd agree this is the most likely interpretation. The language should just be softened a bit.

5) It appears that all the presented analysis was performed at 48 hours APF, would it be possible/informative to examine eyes earlier to try and catch the genesis of these phenotypes? This does not seem essential, but given the variety of described phenotypes it would be good to try and see if one aspect of these defects appears to arise before the others (this also goes to the difficulty in arguing for a particular mechanism, especially FAs vs lateral adhesion).

6) Figure 1 (and possibly elsewhere, but especially Fig. 1) – it would be good to provide a much higher mag view of the clusters (possibly by insets). Defects marked with arrows were hard to see, much less at published dimensions. It’s sometimes hard to see even after really (really!) magnifying the pdf.

7) Where statements on intensity levels are made, it would be good to make a few measurements to improve the level of statistical analysis in the manuscript.

8) Line 312 – wasn’t the Skau et al. paper retracted? Might not be appropriate to cite if so…

9) Not always clear what the n number is referring to (suspect it’s cells, but should be stated).

Minor notes:

Paragraph of lines 74-94 is pretty non-specific, with statements like “the role of E-cadherin and N-cadherin has been explored” (and?) or “X, X, X, and X were also linked to cell shape”. It would be good especially for potentially important factors related to this study (Rho1, Cdc24, cadherins) to describe the exact nature of these findings.

Paragraph of lines 181-194 had more awkward language than others “Of foremost” (?), “plethora”, etc.

First revision

<u>Author response to reviewers' comments</u>

Because of formatting issues, our responses are uploaded as a Supplementary Information file. Please, find them there.

Point by point response to the comments of Reviewer #1:

1. Substantial quantifications are included. As much as possible, the authors should indicate the

© 2023. Published by The Company of Biologists under the terms of the Creative Commons Attribution License (https://creativecommons.org/licenses/by/4.0/).

number of animals quantified. This inclusion is especially important when applying statistical tests, which should be done with N equal to the number of animals rather than N equal to the number of ommatidia or cortical ROIs, a concern relevant to the basolateral F-actin measurements in Fig 6E.

We fully agree with the Reviewer that sample number is important, therefore we already included the relevant information in the original version of the paper (lines 484-485 and 512-513). Now we have extended this by providing a more specific description as to Fig. 7G (formerly, 6E) in the M&M section.

2. To test if the effect on basolateral F-actin is specific, levels of apicolateral F-actin should also be quantitatively compared between control and the double mutants.

We followed this advice, and we provide a quantification for apical actin as well. Based on these measurements, F-actin level appears to be reduced in the basal, lateral and apical levels as well. Because DAAM and FRL are present in all these layers of the eye, it is not a surprising finding. Yet, one can conclude that the concomitant lack of these two formins does not have a specific effect on basolateral (vs apical) actin, and this is now included in the revised text.

3. A underdeveloped aspect of the paper is the effect of the double mutants on lateral cell-cell adhesion. Is local cell-cell adhesion affected or unaffected in addition to the dramatic cell shape changes? Local effects on adhesion could be examined by immunostaining of candidate cell adhesion molecules and/or by TEM of lateral cell-cell contacts.

We believe that our studies clearly revealed that the lateral cell-cell adhesion of the IOCs is affected/partly lost in the double mutants. Presumably, this is best demonstrated in Figure S3B (formerly S2B) where the IOCs are specifically labelled with GFP allowing us to follow their shape and position across the entire retina. Because it might have been difficult to fully appreciate the 3D aspects of this phenotype, we generated a 3D reconstruction of a small group of IOCs based on the confocal images, and a comparison of the wild type and the formin double mutant situation is now provided in Figure 3I. It is important to highlight that these schemes are created by tracing the shape of the individual IOCs across about 80 confocal Z-sections, and this analysis clearly demonstrates the partial lack of cell adhesion between some of the IOCs. Given that this is a critical aspect of the phenotype, we are grateful for the Reviewer to indicate that this point needs further clarification. We are hopeful that the new graphical reconstructions will be highly beneficial to better understand the complex changes induced by these mutations.
4. The side views in Figure S3 G-I are informative and would be good to include in a main figure.

We also think that these panels are informative, however, we felt that they do not fit well with the former main figures. After reconsidering this issue, we propose to move the entire Figure S3 to the main figures.

5. It would be good to confirm that the double mutant eyes are defective in light transmission, using the deep pseudopupil (DPP) assay.

In our understanding the deep pseudopupil (DPP) assay is suitable to detect structural alterations affecting the rhabdomeres, and it is assumed that such alterations cause defects in light transmission. Provided that the formin double mutant eyes clearly exhibit an impaired rhabdomere organization (see for example the sagittal eye sections in former Figure S3, now Figure 4), the DPP assay would not tell more than what we already know and document in the manuscript. Nevertheless, we've been inspired by the comment of this Reviewer, as it remains a highly interesting question what are the functional consequences (if any) of the IOC phenotype reported here. We attempted to address this issue by investigating the visual capabilities of the flies with the well characterized escape response (Card et al., 2008) evoked by light-off stimuli. In this assay only flies with impaired 3-dimensional vision show jump response (e.g. white-eyed flies). In our behavior system the sound volume of wing vibration during the jump-and-flight responses was recorded and analyzed (Fenckova et al., 2019). 5- to 7-day-old flies were subjected to gradually increasing light-off stimuli, that is light was switched off from a default light intensity ranging from 0 to 96 (arbitrary units corresponding to 0 to 13 lux). Each light-off stimuli were administered in 10 iterations and 64 individuals were measured from each genotype. These experiments

© 2023. Published by The Company of Biologists under the terms of the Creative Commons Attribution License (https://creativecommons.org/licenses/by/4.0/).

revealed that except of the control white-eyed Iso31 strain, no other lines showed any escape response (see the graph below). Thus, it appears that the 3-dimensional vision of the formin mutant flies was not impaired enough to provoke a jump reflex. Given that the strongest *DAAM*, *frl* mutant combination could not be analyzed in this assay (due to lethality), while in the adult viable combinations (examined here) more than 60% of the ommatidia are wild type, we interpret these data as an indication that the remaining visual performance is sufficient to control the jump reflex. It follows that more advanced assays would be required to thoroughly investigate the visual capabilities of the formin mutant flies, which is however beyond the scope of this paper.

NOTE: We have removed unpublished data that had been provided for the referees in confidence.

6. The specificity of the in situ immuno-staining with Frl and DAAM antibodies should be confirmed by staining eyes with experimental depletion of Frl or DAAM, or by referencing papers where this test may have been done. Testing specificity with western blots is insufficient.

Specificity of the DAAM antibody was already demonstrated both in immunohistochemistry and Western blot in the Gombos et al., 2015 paper (referred in the M&M section). Specificity of the more lately generated anti-FRL serum was shown in the Toth et al., 2022 paper by Western blots, and here we further confirm it by immunostaining on pupal eyes. These results are now included in Figure S6.

7. Data showing Cdc42 localization should be added or referenced.

Despite a number of functional studies, localization of Cdc42 in the pupal eye was not reported in previous papers. To address the request of the Reviewer, we first probed the Cdc42 antibody generated in the Tepass lab (Harris et al., 2008). Unfortunately, the quality of this old antibody significantly dropped down over the years precluding the detection of a specific expression pattern in the eye. To overcome this obstacle, next we examined the distribution of a ChFP::Cdc42 fusion protein, expressed from an *sqh* promoter driven transgenic construct (published by the Parkhurst lab, Abreu-Blanco et al., 2012). This transgene has previously been successfully used to monitor the subcellular localization of Cdc42 during embryonic development, and here we found that in the pupal eye the ChFP labelled Cdc42 strongly accumulates at the cellular junctions. By analyzing the details, the enrichment at the cell-cell contacts is particularly evident in the IOCs, both in the basal and lateral layers of the eye, which is in good agreement with a role in the regulation of formin activity at the basolateral membranes. A description of the ChFP::Cdc42 expression pattern is now included in the revised paper, and the data is documented in Figure S8.

Point by point response to the comments of Reviewer #2:

A general comment of the Reviewer was the following:
"the authors conclude that these factors are required for lateral adhesion between the interommatidial cells and also play a direct role in setting the basal morphology of these cells. I am not convinced this is the case, based on the data they present in the paper. What is the evidence for lateral adhesion? Figure 3 shows that all interommatidial are present at the apical surface of the tissue. More basally, some are no longer detected, and accordingly, when reaching the basal surface many are missing. This suggest a failure for some of these cells to reach the basal surface - elongation failure? If some interommatidial are not contributing to the basal surface, then this should lead to significant defects in basal surface organization. This does not necessarily involve a direct role for these Formins in regulation the basal cell cortex. Instead, it could be a consequence of defect in cell packing, basally. I think more work is needed to ensure the main conclusion presented in the paper is correct."

Whereas we noticed that it is not trivial to fully appreciate the 3D aspects of the eye phenotype exhibited by the formin double mutants, we believe that our studies clearly revealed that the lateral cell-cell adhesions of the IOCs are affected/partly lost in the double mutants. The strongest evidence for this is shown in Figure 3 where we illustrate how IOC position and shape is altered in the formin mutants. By tracing a few dozens of IOCs individually in confocal Z-sections spanning the entire apical-basal axis of the retina, we made several key observations: (1) the apical cell-cell contacts of the IOCs remain largely normal; (2) in the lateral regions many of the IOCs acquire

© 2023. Published by The Company of Biologists under the terms of the Creative Commons Attribution License (https://creativecommons.org/licenses/by/4.0/).

an irregular shape, and importantly, the lateral adhesion between the SPCs and the TPCs or the bristle cells is lost (or partly lost) on one side of the SPC (meaning that the SPC is laterally attached to one of the two IOC neighbors, but not both); (3) in the most basal layers (at the level of the IOC feet) the rosette pattern is lost due abnormal cell shape and cell-cell contacts between the IOC feet, nevertheless, each IOC foot is attached to the basal lamina (meaning that each IOC extends from the apical till the basal surface, just as in the wild type situation). Based on these observations, the interpretations and speculations raised by this Reviewer appear to be a misunderstanding of the data. To increase clarity, we generated a graphical 3D reconstruction of a representative group of IOCs (entirely based on the confocal Z-sections corresponding to these cells) to better illustrate the major aspects of the phenotype in a series of (relatively) simple cartoons, which are now added to Figure 3. We are hopeful that the new graphical reconstructions will be highly beneficial to better understand the complex changes induced by these mutations.

Specific comments of Reviewer 2:
- A quantitative analysis of the expression patterns for Frl and DAAM, where a co-localization study would be advantageous. This would make it easier to understand where these co-localize and where they do not. FRL seems quite vesicular in the photoreceptors and interommatidial cells?

As suggested by the Reviewer, we quantified the degree of co-localization between FRL and DAAM. Consistent with the rest of the analysis, the measurements were carried out at three layers along the apico-basal axis of the eye. This study revealed the highest level of co-localization in the basal layer (almost exclusively occupied by the IOCs), a somewhat weaker co-localization at the lateral region of the IOCs, and a rather weak overlap in the apical layer. Overall, these quantifications are much in line with our main conclusion as to protein localization: both formins are enriched in the basal and lateral regions of the IOC membranes, but they fail to show a notable overlay in their apical distribution. Results of the quantitative analysis are shown in Figure 6 (formerly Figure 5), and referred in the main text accordingly, whereas a detailed description is provided in the M&M section. In addition, an FRL antibody specificity test is also included (shown in Fig. S6), revealing that the (partly) vesicular FRL signal in the photoreceptors is likely to represent a non-specific background staining.

- Figure 3: the apical membrane of the photoreceptors seems affected. The authors should describe this aspect of the phenotype, if only briefly. It fits with the expression pattern of DAAM, which seems localised in these cells, enriched apically.

Based on these images it is very hard if not impossible to judge the apical membrane of the photoreceptor cells because at this developmental stage those are located somewhere close the middle of the ommatidia and run (nearly) perpendicular to the focal planes shown in this figure. Thus, we assume that the Reviewer might have meant the lateral membrane of the photoreceptors that is visible in panels C-D', and it appears that some irregularities might be present in their actin staining as compared to the highly regular and largely uniform pattern in the wild type. Although we cannot fully exclude the possibility that these differences are real, by looking at many other sections and considering the very significant changes in the cellular pattern, we think that at present it is impossible to unambiguously conclude on this potential component of the phenotype. Because of this uncertainty, we prefer not to comment on this phenotype, as it does not concern the main focus of the paper, even if it indeed fits with expression pattern of DAAM. Moreover, we also note that the 54C-Gal4 controlled IOC-specific expression of DAAM or FRL rescues the eye phenotypes, which is not expected if any of these formins has a function in the photoreceptors.

- To my eyes, it is not possible to separate the basal membrane of the photoreceptors and the membrane of the interommatidial cells- these plasma membranes are apposed. This makes it very difficult to ascertain whether the staining for Frl, DAAM (Fig.5) and F-actin (Fig.6) belong to the photoreceptors or to the interommatidial cells. This is especially problematic since both Formins are expressed in the photoreceptors. Perhaps the authors could make use of the 54CGal4 driver to inhibit frl/DAMM in the interommatidial cells (e.g. DAAM-/-; 54C>frl RNAi) and quantify this genotype?

The Reviewer states that "it is not possible to separate the basal membrane of the photoreceptors and the membrane of the interommatidial cells" as these plasma membranes are apposed. Whereas at some regions the referred membranes indeed run very close (and parallel) to each

© 2023. Published by The Company of Biologists under the terms of the Creative Commons Attribution License (https://creativecommons.org/licenses/by/4.0/).

other, which makes it difficult to discriminate between them, it is important to point out that the shape of the IOCs and the photoreceptor cells is very different from one another that helps to distinguish them. The IOC membranes typically display a straight appearance and they border rectangles (corresponding to the SPCs) or hexagons (the TPCs) or circles (the bristle cell complex), by contrast, the basal membrane of the photoreceptor cells is usually somewhat curvy, and although this membrane seems to abut the IOC membrane at some places, it does not follow it precisely. If we carefully examine the FRL expression pattern in the lateral layers, the straight lines in the region of the IOCs are evident, while the partly diffused, partly dotty pattern in the region of the photoreceptors is most likely a background staining as demonstrated by our antibody specificity experiment (Figure S6 in the revised paper). As compared to this, the DAAM staining in the lateral region not only highlights the lateral membrane of the R cells, but instead of running straight along the IOC membranes, it often has a curved appearance in the vicinity of the IOCs. Because the colocalization analysis (which is now quantified) revealed a significant overlap between the FRL and DAAM staining, we concluded that "*In the lateral region of the eye FRL is mainly found in the cortical membrane of the IOCs, whereas DAAM shows an overlapping accumulation together with an enrichment in the RC membranes*", and we argue that this description is fully supported by the data shown. Yet, we recognize that it presumably was a (too) concise summary, and we extended our description.

Despite the above arguments, we generated DAAM-/-; 54C-Gal4/frl RNAi pupal eyes, as suggested by the Reviewer, and we found that they exhibit very similar ommatidia fusion phenotypes as the $DAAM^{Ex4}$; frl^{59} double mutant combination which is now quantified in Figure S5. This finding, together with the 54C-Gal4 rescue experiments further confirms that FRL and DAAM are only required in the IOCs. Thus, as far as protein functions are concerned, despite the presence of DAAM in the R cells, we conclude that DAAM does not play a non-redundant role in R cell development, and it does not have a redundant role with FRL there either. Nevertheless, a redundant role with another formin cannot be excluded (as discussed in our paper).

- Line 90: Longley & Ready show a requirement for integrin in basal contraction, not in patterning. The authors should modify their statement accordingly.

Modified accordingly.

- Line 163: we expressed …/… in the developing IOCs by 54C-Gal4: Figure 3 does not show this.

A reference for the appropriate figure is now included.

- Line 194: " misoriented rhabdomeres": what do the authors mean by this?

The text was revised to increase clarity.
- Scale bars are missing in some figures, for example Fig.5.

The missing scale bars are now added.

- Line 305: "we conclude that cdc42 controls the activity of both Formins: This conclusion is not very solid as it is based on the finding that the phenotypes are similar and that they interact genetically. To back up this conclusion, the authors need to show evidence that these Forming act downstream of cdc42.. For example, they could express Frl or DAAM in cdc42 retinas and assess whether this rescues the cdc42 phenotype. They could also quantify the expression of Frl and DAAM in the cdc42 retinas, of when using the cdc42 RNAi.

We agree the Reviewer that based on our findings it is was inappropriate to firmly conclude that "Cdc42 controls the activity of both formins", therefore we toned down our statement. However, we also need to note that besides the phenotypic similarities and the genetic interactions, our conclusion is strongly supported by the knowledge that the major activators of the DRF family formins are the Rho GTPases, and a specific interaction has already been reported for Cdc42 and DAAM, as well as for Cdc42 and FRL. The reviewer proposed to test whether the expression of FRL or DAAM rescues the cdc42 LOF phenotype. This experiment has been carried out (see the chart below), but no rescue was detected. In our interpretation this is not a surprising data, as if we assume that Cdc42 is required for formin activation, it is not expected that the lack of an

© 2023. Published by The Company of Biologists under the terms of the Creative Commons Attribution License (https://creativecommons.org/licenses/by/4.0/).

activator can be compensated by the overexpression of their target molecules that are autoinhibited. Thus, this result is in line with our model, although we think that this experimental design is not appropriate to examine whether the formins act downstream of Cdc42 in the IOCs. In our view, to test the epistatic relation, it would be more suitable to ask whether the effect of activated Cdc42 can be suppressed by loss of the formins. Unfortunately, this is not a feasible experiment because the expression of activated Cdc42 has a drastic effect on the shape and differentiation of the retinal cells, which would prevent the analysis of the ommatidia fusion phenotype.

As to the quantification of Frl and DAAM in the *cdc42* mutant retinas. The Rho GTPases are not known to regulate the expression level of the formins, and they are also not known to be required for their subcellular accumulation, instead they were reported to bind the formins directly and relieve their autoinhibition. For these reasons, this quantification seems rather superfluous.

NOTE: We have removed unpublished data that had been provided for the referees in confidence.

- They use many RNAi lines for their work: how here these RNAi lines validated to ensure they to not come with off-target effects?

The “many RNAi lines” mentioned by the Reviewer appear rather countable to us, as we used only five of them, all listed in the M&M. As to specificity, in the cases of FRL RNAi (BL#32447), Cdc42 RNAi (BL#37477) and DAAM RNAi (#39058) the silencing resulted in highly similar phenotypic effects as the ones observed with the classical alleles of the corresponding genes. In the case of the DAAM PD RNAi line much the same was observed and reported by Chougule et al., 2020 (although using another tissue). As a geneticist, I can hardly think of a more convincing evidence to exclude the off-target effects. The last line we used was *zip* RNAi (BL#36727), and in this case we tested an additional line (BL#65947) that exhibited a very similar effect as the former one (data is now included in Figure 9). Thus, we think that compelling evidence are provided to rule out the off-target effects.

Point by point response to the comments of Reviewer #3:

1) As a potential approach to better get mechanism, what do the cadherins look like in these backgrounds? This would seem to allow for a better argument for a FA vs cadherin mechanism.

There are two classical cadherins known to be expressed in the retina, Drosophila E-cadherin and N-cadherin. Data for Ecad has already been provided in the original version of the paper, and to follow the suggestion of the Reviewer, we now provide data for Ncad as well (shown in Figure S1). Much as for the case of Ecad, the lack of FRL and DAAM does not affect the expression pattern and localization of Ncad. Given that Ncad is not expressed in the IOCs, this result is entirely consistent with our expectations.

Regarding the potential “FA vs cadherin mechanisms” mentioned by the Reviewer, we would like to clarify that based on our observations the basal side of the IOCs remains attached to the basal lamina. In agreement with this, the integrin-based FA complexes are clearly present in the basal region. This was shown by a ¤-integrin staining in the original version (Figure S4), and we have further confirmed it by adding a Talin staining (see revised Figure S4). Because many of the IOCs and the grommets acquire an abnormal shape and position, at first glance it might appear that in the absence of the formins the staining patterns of the FA proteins are altered, however, a more careful look reveals that in the *DAAM; frl* double mutants the FA complexes still enrich along the cell borders and at the grommets, much alike as in the wild type situation. Therefore, we think that the FA based mechanisms are unlikely to have a significant contribution. Because lateral adhesion of the IOCs is one of the major defect observed in the mutants, molecules involved in lateral cell adhesion (including Ecad, known to be present in the lateral membrane of the IOCs) are among the best candidates for further mechanistic studies.

2) Similarly, could overexpression of Zipper, Sqh, or activated Rho be done to see if FAs are weakened, or if particular regions of retinal organization appear to be differentially affected?

We tested the overexpression of Sqh, and we found no effect on eye-development, whereas for Zipper there is no available UAS line in the public stock centers (and we also failed to obtain such a line from other sources). The expression of activated RhoA prevented normal pattern

© 2023. Published by The Company of Biologists under the terms of the Creative Commons Attribution License (https://creativecommons.org/licenses/by/4.0/).

formation in the eye. Hence, this set of study was not particularly informative, and we did not include it in the revised version.

3) Line 213, “The ommatidium fusion phenotype and the retinal floor defects observed in the DAAMEx4; frl59 mutants indicated a formin requirement in the IOCs”. This does not seem accurate (“IN the IOCs”); at this point in the manuscript it could still be through non-autonomous effects.

Because the phenotypes described in the previous sections of the paper clearly affected the IOCs, and the formins are cytoplasmic proteins, we think it a perfectly logic and appropriate assumption that these formins are required in those cells (without having a non-cell autonomous role). Obviously, the Reviewer is correct to say that up to that point of the paper we did not present a formal proof for this, but we don’t think that the phrase “indicated” would imply a formal proof. For this reason, we suggest to stay with the original version.

4) Line 305-306 - it can’t be formally concluded that Cdc42 controls formin function based off of these experiments (absent Frl or DAAM staining or epistasis), though I'd agree this is the most likely interpretation. The language should just be softened a bit.

Done.

5) It appears that all the presented analysis was performed at 48 hours APF, would it be possible/informative to examine eyes earlier to try and catch the genesis of these phenotypes? This does not seem essential, but given the variety of described phenotypes it would be good to try and see if one aspect of these defects appears to arise before the others (this also goes to the difficulty in arguing for a particular mechanism, especially FAs vs lateral adhesion).

This is undoubtedly a very exciting question. We attempted to address this issue by using fixed samples, as well as live imaging methods. The major conclusion we could make is that in fixed samples the ommatidia fusion phenotype can be detected as early as 20 hours APF (see image below). However, the presence of the uncommitted surplus IOCs (which are not eliminated yet) and the overall lack of such a clear pattern that forms by 40-42 hours APF, prevented us from making solid conclusions as to the order of the events. As to live imaging, the major limitation is that it is only the apical region (and the apical junctional areas) that can be imaged with sufficiently high resolution. By carrying out this experiment, we could confirm that the apical junctions form in a highly similar manner as in wild type, however, the thickness of the tissue prevented of high quality imaging of the lateral and basal layers of the developing pupal eye. Because of the lack of further mechanistic insights gained by these studies, we propose to leave them out from the revised version.

NOTE: We have removed unpublished data that had been provided for the referees in confidence.

20 h APF pupal eyes of the indicated genotype, stained for actin and GFP. Note the emerging hexagonal pattern of the IOCs (marked in green) in the control flies (A). As compared to that, in the formin double mutants the hexagonal lattice is less evident (B), because some IOCs acquire an abnormal shape and/or position.

6) Figure 1 (and possibly elsewhere, but especially Fig. 1) - it would be good to provide a much higher mag view of the clusters (possibly by insets). Defects marked with arrows were hard to see, much less at published dimensions. It’s sometimes hard to see even after really (really!) magnifying the pdf.

A major aspect of the ommatidia fusion phenotype is that it involves at least two (but typically several) neighboring ommatidia. For this reason, full appreciation of the defects is only possible by showing an eye field with a group of ommatidia. We tried to minimize the size of that field, but we think that placing higher mag insets would make Figure 1 (which is already quite big) completely overcrowded. Nevertheless, we changed the color of the arrows to help their visibility. In addition, we note that the former Figure S3 (which became a main figure in the revised version) contains higher mag images with fewer ommatidia than Figure 1, and it hopefully helps to see better.

© 2023. Published by The Company of Biologists under the terms of the Creative Commons Attribution License (https://creativecommons.org/licenses/by/4.0/).

7) Where statements on intensity levels are made, it would be good to make a few measurements to improve the level of statistical analysis in the manuscript.
A more thorough quantitative analysis is now presented in Figure 6 (formerly Figure 5) and Figure 7 (formerly Figure 6).

8) Line 312 – wasn't the Skau et al. paper retracted? Might not be appropriate to cite if so…

Indeed, a Skau et al., 2016 paper has been retracted, but that is another paper, not the one we cite here.

9) Not always clear what the n number is referring to (suspect it's cells, but should be stated).

It is a fair comment. With "n" we referred to the number of ommatidia that was counted, and this is now indicated in the revised paper. In addition, the M&M section contains further descriptions as to the number of eyes examined and of the number of biological replicas.

Minor notes:

Paragraph of lines 74-94 is pretty non-specific, with statements like "the role of E-cadherin and N-cadherin has been explored" (and?) or "X, X, X, and X were also linked to cell shape". It would be good, especially for potentially important factors related to this study (Rho1, Cdc24, cadherins) to describe the exact nature of these findings.

The text was modified to provide more specific descriptions.

Paragraph of lines 181-194 had more awkward language than others "Of foremost" (?), "plethora", etc.

We've tried to improve the language of this paragraph.

<u>Second decision letter</u>

MS ID#: DEVELOP/2023/201713

MS TITLE: FRL and DAAM are required for lateral adhesion of the interommatidial cells and patterning of the retinal floor

AUTHORS: Gabriella Gazso-Gerhat, Rita Gombos, Krisztina Toth, Peter Kaltenecker, Szilard Szikora, Judit Biro, Eniko Csapo, Zoltan Asztalos, and Jozsef Mihaly

I have now received all the referees reports on the above manuscript, and have reached a decision. The referees' comments are appended below, or you can access them online: please go to BenchPressand click on the 'Manuscripts with Decisions' queue in the Author Area.

The overall evaluation is very positive and we are happy to publish your manuscript in Development. Before we can proceed though, could you please correct the typos mentioned by referee 2?

<u>Reviewer 1</u>

Advance summary and potential significance to field

The authors have effectively addressed most of my comments with new data and text revisions which have improved the manuscript. Visual assays were attempted but technical issues prevented clear conclusions. The lack of this information does not affect the major conclusions of the paper.

© 2023. Published by The Company of Biologists under the terms of the Creative Commons Attribution License (https://creativecommons.org/licenses/by/4.0/).

Overall, the paper is substantial and advances the understanding of cytoskeletal regulation controlling the 3D morphogenesis of the insect compound eye.

Comments for the author

Further proofreading for grammar issues and typos is warranted. A few examples are below.

294 "Whereas, we found no significant differences in the formin mutant eyes with regard to apical actin organization (Fig. S7), quantification on fixed samples showed a reduction in apical actin level (Fig. 7A,B,G)."

313 "regulated by Rac and Cdc42 but Rho1 in the embryonic CNS"

351 "The arthropod compound eye is arguably the most broadly used light sensing organ of the living organisms."

Reviewer 2

Advance summary and potential significance to field

I think that overall, the manuscript has improved substantially, and the authors' main conclusion and working model are better supported. This work should be of broad interest the the community of cell and developmental biologists.

Comments & Clarifications:
- The 3D segmentation now provided, does help as it shows that the IOC do not maintain contact laterally. I think that my original point remains, though: whether this is due to a lack of adhesion (i.e., lateral intercellular adhesion system?) remains to be established—perhaps as a follow-up work.

Original comment: To my eyes, it is not possible to separate the basal membrane of the photoreceptors and the membrane of the interommatidial cells…/… Confocal microscopy imaging of two cell populations with apposed plasma membranes does not allow to determine which plasma membrane of the two apposed cells contains a surface receptor. The DAAM-/-; 54C-Gal4/frl RNAi does help here and ensures that the main conclusion is better supported by direct experimental evidence.

- They use many RNAi lines for their work: how here these RNAi lines validated to ensure they to not come with off-target effects? Unfortunately, phenotype similarity does not allow to rule out off-target effects.
There are several ways 3D patterning of a group of cells can be affected in a similar way (different genotypes with similar phenotypes). Properly controlling RNAi lines that have not been published before is important and in my opinion, it should be a gold standard in genetics.

Comments for the author

I would suggest to ensure the unpublished RNAi are specific (flip-out clone stained with AB and rescue assay if possible); or else switch to published, validated lines. This is however only a suggestion and if the authors feel this is not necessary then, this is fine with me.

Reviewer 3

Advance summary and potential significance to field

The authors have taken the reviewer critiques seriously and attempted to seriously address them. They have improved the quantitation and statistics in the revised manuscript, as well as examined numerous other genetic backgrounds and temporal analyses. The new analysis performed for the revision is extensive. In my opinion, the manuscript is appropriate for publication in Development. Congrats to the authors on a nice study!

© 2023. Published by The Company of Biologists under the terms of the Creative Commons Attribution License (https://creativecommons.org/licenses/by/4.0/).

Comments for the author

The authors have taken the reviewer critiques seriously and attempted to seriously address them. They have improved the quantitation and statistics in the revised manuscript, as well as examined numerous other genetic backgrounds and temporal analyses. The new analysis performed for the revision is extensive. In my opinion, the manuscript is appropriate for publication in Development. Congrats to the authors on a nice study!

Second revision

Author response to reviewers' comments

List of the corrections made in the sentences mentioned by Reviewer 1:

294 "Whereas, we found no significant differences in the formin mutant eyes with regard to apical actin organization (Fig. S7), quantification on fixed samples showed a reduction in apical actin level (Fig. 7A,B,G)."

This sentence has been modified as follows:
Whereas we found no significant differences in the formin mutant eyes **concerning** apical actin organization (Fig. S7), **quantitative analysis revealed a decrease in the level of apical actin** (Fig. 7A,B,G).

313 “regulated by Rac and Cdc42 but Rho1 in the embryonic CNS”

This sentence has been corrected as:
“regulated by Rac and Cdc42 but **not** Rho1 in the embryonic CNS”

351 "The arthropod compound eye is arguably the most broadly used light sensing organ of the living organisms."

This sentence has been modified as follows:
The arthropod compound eye is **widely recognized as the most extensively utilized light-sensing organ found** in living organisms.

Third decision letter

MS ID#: DEVELOP/2023/201713

MS TITLE: FRL and DAAM are required for lateral adhesion of the interommatidial cells and patterning of the retinal floor

AUTHORS: Gabriella Gazso-Gerhat, Rita Gombos, Krisztina Toth, Peter Kaltenecker, Szilard Szikora, Judit Biro, Eniko Csapo, Zoltan Asztalos, and Jozsef Mihaly
ARTICLE TYPE: Research Article

I am happy to tell you that your manuscript has been accepted for publication in Development, pending our standard ethics checks.

© 2023. Published by The Company of Biologists under the terms of the Creative Commons Attribution License (https://creativecommons.org/licenses/by/4.0/).
